# Planned synchronization for multi-robot systems with active observations

**DOI:** 10.1007/s10514-025-10225-4

**Published:** 2025-12-24

**Authors:** Patrick Zhong, Federico Rossi, Dylan A. Shell

**Affiliations:** 1 Texas A&M University, College Station, TX 77840 USA; 2 Jet Propulsion Laboratory, California Institute of Technology, Pasadena, CA 91109 USA

**Keywords:** Cooperative plans, Active joint-perception, Planned communication, Re-scheduling under uncertainty

## Abstract

An important class of robotic applications involves multiple agents cooperating to provide state observations to plan joint actions. We study planning under uncertainty when more than one participant must proactively plan perception and/or communication acts, and decide whether the cost to obtain a state estimate is justified by the benefits accrued by the information thus obtained. The approach we introduce is suitable for settings where observations are of high quality and they—either alone or along with communication—recover the system’s joint state, but the costs incurred mean this happens only infrequently. We formulate the problem as a type of Markov decision process (mdp) to be solved over macro-actions, sidestepping the construction of the full joint belief space, a well-known source of intractability. We then give a suitable Bellman-like recurrence that immediately suggests a means of solution. In their most general form, policies for these problems simultaneously describe (1) low-level actions to be taken, (2) stages when system-wide state is recovered, and (3) commitments to future rescheduling acts. The formulation expresses multi-agency in a variety of distinct practical forms, including: one party assisting by providing observations of, or reference points for, another; several agents communicating sensor information to fuse data and recover joint state; multiple agents coordinating activities to arrive at states that make joint state simultaneously observable to all individuals. Though solved in centralized form over joint states, the mdp is structured to allow decentralized execution, under some assumptions of synchrony in activities. After providing small-scale simulation studies of the general formulation, we discuss a specific scenario motivated by underwater gliders. We report on a physical robot implementation mocked-up to respect these same constraints, showing that joint plans are found and executed effectively by individual robots after appropriate projection. On the basis of our experience with hardware, we examine enhancements to the model that address nonidealities we have identified in practice, including the assumptions regarding synchrony.

## Introduction

A fundamental challenge in designing, programming, and optimizing distributed systems is that the overall state will typically not be known to all the constituent elements at all points in time. In multi-robot systems, this challenge is especially acute owing to added uncertainty, born of imperfect actuation and sensing, and intermittent, unreliable, or sparse communication. In this paper we, thus, consider the problem of planning under uncertainty for robots that, as part of their efforts to achieve useful ends, might actively plan and execute actions in order to determine system state. In multi-robot systems this will typically include cooperative peer observations, synchronization, communication activities for fusion of data obtained by different agents, or mutual inter-robot measurements. These activities often bear a cost and, consequently, systems must reason about whether better knowledge of system state improves task performance enough to justify the effort to obtain such information. Particularly interesting are cases where a degree of uncertainty can and should be tolerated in specific contexts of the execution. We provide an approach to handle such cases, with a focus on enabling algorithms to determine when and how to allow looser coupling between the robots.

This paper’s key perspective is to focus specific attention on those instants in time when action-executing elements of the system have precise knowledge of state. There is a class of practical robot problems in which such instants will arise repeatedly: part of the system’s design, or the crafting of agent-to-agent interactions, or both, can ensure such instants do arise, even if only sporadically. Under our formulation of the problem, this leads to plans that include state observation, check-in, and/or synchronization steps as actions. By specifying the triggering of these actions, plans encode when future information will be available, expressing facts about *subsequent* knowledge of state.

To elaborate further, some specific examples will be helpful.

### Motivating scenarios

The formulation we consider admits several variants of multi-agency. The following two practically motivated examples suffice to draw out commonalities, and will aid the upcoming discussion.

#### Example 1

**Fig. 1:**
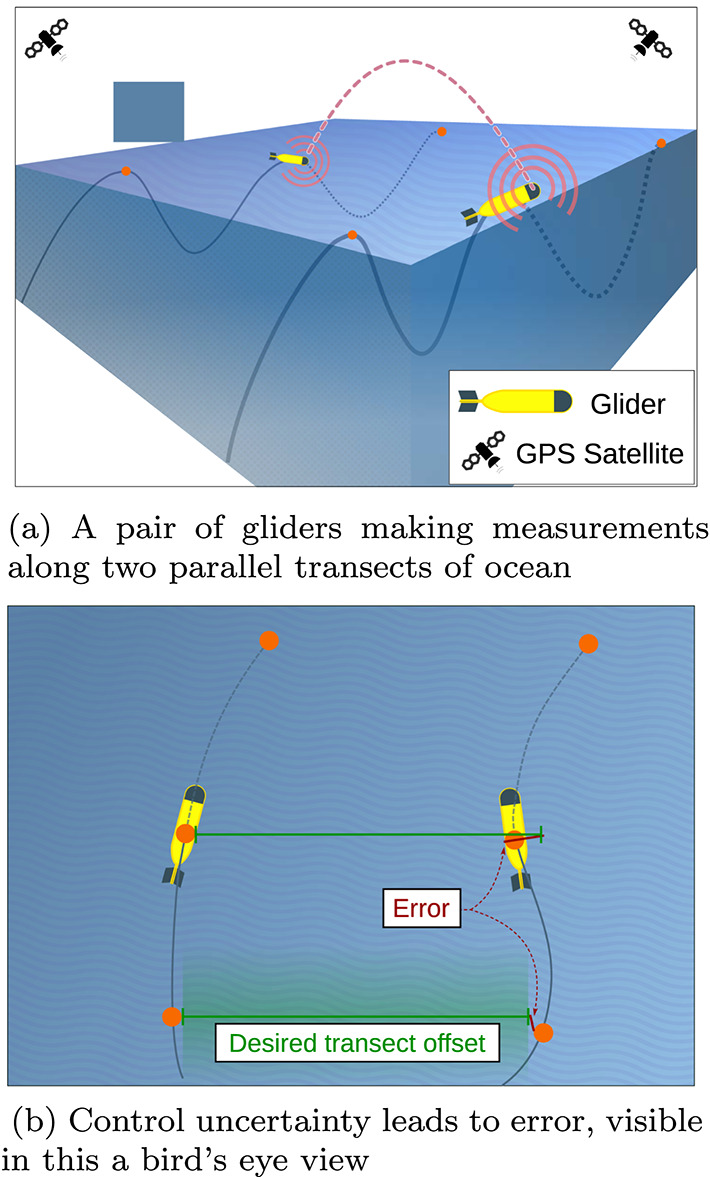
Ocean sampling. A pair of underwater gliders are tasked with making near-simultaneous measurements with a pre-defined spatial offset within a region of interest, employing the characteristic sinking/ rising pattern of locomotion. The devices can communicate and obtain reliable position estimates only once surfaced (points marked pictorially in orange). Observations are costly and, as varying conditions affect the reliability of their dead-reckoning, they determine when to check-in next based on their relative error

(Ocean sampling) As depicted in Fig. 1, consider a situation with a pair of underwater gliders sampling a transect of ocean by tracing parallel tracks, with a pre-defined offset distance between them. The figure shows how these devices move: they dive and float by adjusting their buoyancy and use hydrofoils to turn vertical forces into translational movement. Individual gliders only have access to precise pose measurements in the global reference frame at the surface, and challenges imposed by underwater attenuation mean that communication is all but infeasible except at the surface. The gliders’ predominant mode of operation involves being deprived of sensing and communication for long periods of time, punctuated by intermittent moments with availability of high-quality state sensing and communication. Once surfaced, they determine how much their separation has deviated from the desired offset, what actions will help correct this error, and —more subtly—how deep to dive before resurfacing in order to communicate again. Given the energy required to use the gps receiver and communication hardware, longer sequences with deeper dives between re-surfacing represent an energy saving. On the other hand, sparser pose estimates mean greater between-track error, potentially tainting the gathered data.

While the previous example with homogeneous robots exhibits a high degree of symmetry, the next example does not.

#### Example 2

**Fig. 2:**
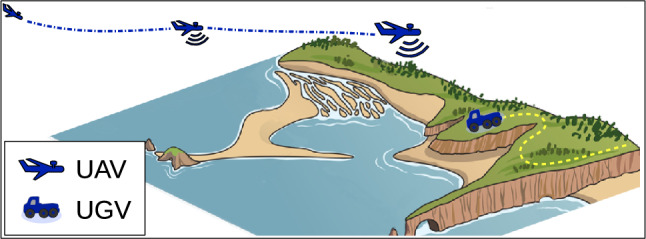
UAV/UGV teaming. A ground vehicle (ugv) traverses complex terrain with actions subject to uncertainty. Fixed-wing uncrewed aerial vehicles (uavs) broadcast their positions to help the ugv localize by ground-based observation

(uav/ugv teaming) Consider the situation depicted in Fig. 2, where an uncrewed ground vehicle (ugv) is tasked with traversing a challenging, potentially contested, gps-denied environment. One or multiple fixed-wing uncrewed aerial vehicles (uav) help the ugv localize itself by periodically flying over the theater and broadcasting their own positions, akin to a gps satellite; by observing the location of a uav relative to itself (while remaining invisible to both the uav and to potential hostile parties), and comparing the uav ’s relative location with the absolute location it broadcasts, the ugv can localize itself accurately. The uav ’s minimum turning radius; energy consumption; and risk of detection all introduce a cost to the localization process, and encourage a less frequent overflight schedule. At certain junctures, the ugv may wish to request changes in the overflight schedule, e.g., more frequent overflights as it approaches highly challenging terrain. This, however, requires communication from the ugv to the uav, which incurs additional cost due to the risk of detection; accordingly, schedule re-plans are likely to be rare in an optimal schedule.

In both examples, interaction between agents is needed in order to determine the system’s state: observations are either direct simultaneous measurements of joint system state, or are sufficient when combined via communication. For a range of times, global system state will not be precisely known: in Example 1, only at those specific points where the gliders surface and make observations will the state be identified; for Example 2, the pose of the ugv is recovered only at the overflight times. In the underwater glider case, when the state is known, it will be known to all the agents that compose the system. When the pose of the ugv is determined, by contrast, it is ordinarily known only by the ugv—with the exception being if other communication to the uav occurs as, for instance, in requesting changes to the overflight schedule.

The interplay of the agents is structured around forming commitments, sticking to them, and then repeating the process. A glider in Example 1 has limited knowledge of the other’s pose in the time elapsed since their previous check-in, but their re-surfacing is coordinated owing to decisions made when last at the surface. The ugv of Example 2 has a schedule of uav overflights and uses this to know when state can next be determined; these future commitments change only upon explicit rescheduling, should that happen to occur. In both instances, the formation of new commitments is affected by current knowledge of the global state—fresh information alters subsequent action. If, battered by uncertainty, the system has been driven into a precarious position, in addition to the selection of more cautious actions, it is likely worth paying the cost of gaining information/of communicating often. Conversely, if the realized randomness has been mainly felicitous, the system may now be in the fortunate position of requiring fewer check-ins.

Finally, note how information is obtained *actively*—the robots in the system plan and, through fully distributed execution, together realize an observation-generating process. Although couched in terms of observations of state, this also expresses a form of the central problem in sequential decision-making for multi-robot systems: deciding when to communicate. In the absence of continuous communication, individual robots must explicitly account for uncertainty in the other robots’ states and actions, which is challenging from a computational standpoint. On the other hand, in typical deployed robotic systems (and circumstances underlying the settings in Examples 1 and 2), continuous communication is resource-expensive; may reveal the agents’ presence and position to hostile third parties; and achieving sufficient bandwidth may be entirely infeasible in certain environments (e.g., underwater or in cave environments). These considerations align with broader interest in the design of multi-agent systems that explicitly reason about communication, and plan to communicate sparingly only when advantageous.

### Contribution

This article offers the following contributions. First, we formalize the problem of planning with scheduled state synchronization as a type of Markov decision process (mdp). The type of optimal decision-making policy for the problem is a complex object, involving three nested considerations, namely, (i) optimizing the individual robots’ actions (accounting for the uncertainty in other robots’ states and actions) for a given check-in schedule, (ii) optimizing the check-in schedule over a given horizon, and (iii) optimizing the times at which the check-in schedule can be revisited. We are not aware of coordination plans of comparable character within the literature. For multi-robot problems, the treatment we present will also exploit two orthogonal species of composition (macro–joint actions are formed that aggregate across agents and across time). Second, we provide additional insight into special cases of the problem, namely, when the rescheduling cost is zero (which allows robots to re-plan the check-in schedule at every check-in), and when the rescheduling cost is infinite (which forces robots to commit to a check-in schedule before deployment). The latter case is explored, briefly, in a multi-objective setting as well. Third, we study the problem through numerical and hardware experiments. Lastly, inspired by the insights derived in the hardware experiments, we examine and quantify the effect of non-idealities such as imperfect observations and communication delays.

The ideas in this article extend those appearing in two specific, precursory conference papers: Zhong et al. (2024) and Zhong et al. (2023). The former paper has new schedules computed at every stage; the latter paper considers the case without any rescheduling. These correspond, respectively, to where rescheduling operations are gratis versus infinitely costly—in this sense, the prior work characterizes points at extreme opposite ends of a spectrum parameterized by the cost of forming new schedules. The work in those prior conference papers was thought to have treated qualitatively different problem settings, lacking any explicit notion of scheduling as an activity which might incur a cost. The formulation we present here not only generalizes the two pieces of prior work, but has unified them by established that there is (as represented by differing rescheduling costs) a meaningful quantitative spectrum in between. The rest of the paper is as follows. After a brief discussion of the most closely related work in Sects. 2 and 3 gives our formulation of the problem. Then, Sect. 4 provides algorithms to solve the problem formulation, and studies in more detail the two extreme cases of no rescheduling cost and infinite rescheduling cost. Section 5 presents a hardware demonstration and studies the resulting policy in detail. The effect of several non-idealities identified in the hardware demonstration is quantified in Sect. 6. Finally, we draw our conclusions and lay out directions for future research in Sect. 7.

## Related work

Observations that are sparse can be treated via a partially-observed Markov decision process (pomdp); for a recent survey of the state of the art in the robotics context, see Lauri et al. (2023). And, when dealing with multiple agents, there exists work on decentralized pomdps  (Amato et al., 2019). These approaches, reviewed recently in Zhang et al. (2021), seek approximate search algorithms to handle the enormous space of beliefs arising from partial observability and the combinatorial nature of the joint-action space in Dec-pomdps. To help improve tractability, pomdps can address the sub-problems of *when* to localize (Williams et al., 2024) and *what* to communicate (Roth et al., 2005), but treating only part of the coordination concern is, inherently, limited. Critically, the pomdp formulation in full generality also overlooks structure ripe for exploitation: the formulation we present has no explicit representation of beliefs as, at the synchronization points, beliefs project back to the state space. Further, in contrast, we can compute exact solutions under our model.

Also within the literature, decentralized mdps utilize local observations from distributed agents, such as in Bernstein et al. (2002). In that model, as also in our case, when observations arrive their union provides the joint system state. Unlike that model, we are considering sparse observations and must decide when observations ought next to occur. Worth further mention, a set of approaches consider decentralized reinforcement learning; one recent example having agents communicate intent appears in Yang et al. (2023). Those authors employ sampling to learn the transition model, whereas our approach is model-based.

The work of Marcotte et al. (2020) optimizes communication policies, selecting observations to share between agents in an online fashion. Also, decentralized planning via tree search has been effectively used to improve multi-robot active perception (Best et al., 2019).

The sensing model we employ is sparse but provides high-quality observations (i.e., full state) when information is obtained; the idea of studying pomdps where it is optimal to obtain perfect state information at finite intervals is examined by Hansen (1994), wherein he determines a sensing rate at run-time; his formulation (and even notation) turns out to be nearly identical to the approach we outline for zero scheduling cost. In Hansen (1997), he terms these mdps with observation costs, and points out that that there are early examples (going back more than 50 years) of inspection tasks that fit this model well. These are part of the broader question of selective sensing (e.g., see early work in Langley (1997) and references therein) which ultimately is a specific form of active perception (Bajcsy et al., 2018). Another extremely similar line, again directly related as full state information can be obtained for a cost, is dubbed the oracular pomdp model (Armstrong-Crews & Veloso, 2008). These prior works have considered dynamic programming and policy iteration methods as well as online approaches (e.g., with look-ahead heuristics). An only slightly different method of limiting sensor use that allows for dynamic observations is the use of mdps for self-triggered control (Huang & Zhu, 2021) wherein a subpolicy determines when sensor inputs will be needed next. That approach, however, relies on the reduction of the action space to time-homogeneous macro-actions which constantly repeat a single action. Recent work of Reisinger and Tam (2024) suggests this may be expanded to a limited set of time-inhomogeneous macro-actions, but they are to be prescribed *a priori*. Other recent work, such as that of Kapoor and Nair (2025), continues to explore a variety of techniques and approximations for mdps with observation costs.

In sum, from Hansen’s work forward, there is a rich wealth of practical approximation and heuristic ideas to help improve planning performance. But because our setting supposes that altering a schedule may incur costs, some generalization of these concepts will be required to handle the full formulation described herein. Nevertheless, these prior works serve as excellent sources of inspiration and potential insight for future algorithmic enhancements. It is worth also pointing out that despite the existence of such prior work on solutions for the special case of free rescheduling, the suitability of such models (even restricted to this special case) for the multi-agent case seems to have been overlooked. Indeed, the idea that agents might have policies causing them to act so as to observe other agents, or policies demanding they engage in communication, or otherwise establish and synchronize shared state, is not a contrived one. Not only will the study in Sect. 5 illustrate the naturalness of this fit, but Sect. 6 then goes on examine additional uncomplicated modifications that further improve how to model multi-agency.

## Problem formulation

### Preliminaries and notation

We assume the reader is familiar with a standard (centralized) Markov decision process; established references are Bertsekas (2019); LaValle (2006); Puterman (1994). In the classic discrete time model, at each stage, some observation discloses the state of the underlying Markov process and the decision maker chooses an action, the process evolves according to it, and then this repeats. The decision maker has the objective of minimizing some cost; we treat the infinite horizon case with the usual cumulative discounted costs (but will revisit this and consider a multi-objective perspective in Sect. 4.3.1).

We will write $\mathbb {R}$ for real numbers, $\mathbb {N}$ for positive integers, and $\mathbb {N}_{\ge 0}= \mathbb {N}\cup \{0\}$. For maximal concision, sequences will be written just as $x_0 x_1 x_2 \!\ldots x_m$. The length of a sequence $\mathbf{y} = y_0 y_1 \!\ldots y_k$ is given as $|\mathbf{y}| = k+1$. For any non-empty set *X*, we use the Kleene star, $X^*$, to denote the set of all finite sequences of elements of *X*. The Kleene plus is defined as $X^+= \big \{\mathbf{x} \in X^*: 1 \le |\mathbf{x}|\big \}$. Their difference is $\varepsilon$, the ‘empty’ sequence. The notation $X^\omega$ represents the set of sempiternal sequences of elements of *X* and a bar will be used to show infinite repetition: $x_0 x_1 \overline{x_2x_3} = x_0 x_1 x_2 x_3 x_2 x_3 x_2 x_3\!\ldots \in X^\omega$.

We make use of a slight abuse of notation that lightens the presentation significantly. In several places, we will consider relations and expressions involving optimization expressed via a ‘$\min$’ operator; we shall write ‘$\mathop {\mathrm {arg\,min}}\limits$’ when we wish to refer to a minimizer, even when —somewhat unconventionally—the reader must unpack the expression and locate the ‘$\min$’ and its argument themselves.

### Scheduling and composition of macro-actions across time

Our model has three notions of cost: the standard cost associated with the execution of some actions (shortly to be denoted $C_{{\mathrm{exc}}}$), the cost of obtaining a state observation (ditto $C_{{\mathrm{obs}}}$), and the cost of scheduling observations ($C_{{\mathrm{sch}}}$). The last of the three is, perhaps, the most subtle; one might think of it as accounting for the work of negotiating upcoming parts of a schedule. (In Example 2, there is specific communication from the ugv to the uav that must happen, and it has some cost).

Those necessary additions, yield our definition:

#### Definition 1

(mdpans) A *Markov Decision Process with Actively Negotiated Schedules* is a tuple $$\langle S, A, T, $$$$C_{{\mathrm{exc}}}, $$$$C_{{\mathrm{obs}}}, $$$$C_{{\mathrm{sch}}}, $$$$\gamma , \xi ^0\rangle $$ where

○ $S = \{s_1, s_2, \dots , s_{|S|}\}$ is the finite set of states;
○ $A = \{a_1, a_2, \dots , a_{|A|}\}$ is the finite set of actions;
○ $T: S\times A \times S \rightarrow [0,1]$ is the transition dynamics, or transition model, describing the stochastic state transitions of the system, assumed to be Markovian in the states, where $\forall t, P(s^{t+1} = s' | s^{t} = s, a^{t} = a) = T(s',a,s)$;
○ $C_{{\mathrm{exc}}}: S\times A\rightarrow \mathbb {R}$ is the execution cost function, prescribing that the cost $C_{{\mathrm{exc}}} (s,a)$ is incurred by taking action *a* from state *s*;
○ $C_{{\mathrm{obs}}}: S \rightarrow \mathbb {R}$ is the check-in cost function, with $C_{{\mathrm{obs}}} (s)$ incurred when a state observation is taken whilst in state *s*;
○ $C_{{\mathrm{sch}}}: S \rightarrow \mathbb {R}$ is the scheduling cost function, with $C_{{\mathrm{sch}}} (s)$ being incurred in negotiating a schedule for next set of check-ins with the system in state *s*;
○ $\gamma \in [0,1)$ is the discount factor;
○ $\xi ^0:S \rightarrow [0,1]$ is the initial state distribution.

For convenience, as we will be interested in sequences of actions of differing length, we define $\mathbf{A}= A^1 \cup A^2 \cup \cdots = A^+$.

Intuitively, though elementary actions *A* are provided, the decision maker selects sequences of such actions (the elements of $\mathbf{A}$). These are essentially open-loop macro-actions. Each macro-action has a transition dynamics and cost derived from the atomic (i.e., single-step) versions. We use the operations of transition and cost composition to form $\mathbf{T}$ and $\mathbf{C} _{{\mathrm{exc}}}^{\gamma }$ from these more elementary actions *A*:

#### Construction 1

(Transition composition (Rossi & Shell, 2022)) Given elementary actions *A* and their associated transitions $\,T: S\times A \times S \rightarrow [0,1]$, the macro-action transition model is the function $\mathbf{T}: S\times \mathbf{A}\times S \rightarrow [0,1]$ defined as

$$\begin{aligned} \mathbf{T}\left( s',\mathbf{a}, s\right)&\;=\,\; \mathbf{T}\left( s',a_1 a_2 \!\ldots a_{|\mathbf{a}|}, s\right) \\&\;\;= \hspace{-4.0ex}\sum _{\begin{array}{c} s_0s_1 \!\ldots s_{|\mathbf{a}|}\in S^{|\mathbf{a}|+1}\\ \!\!\!\!\!\!\text {where } s_0 = s\\ \!\!\!\!\!\text { and } s_{|\mathbf{a}|} = s' \end{array}} ~ \prod _{i=1}^{|\mathbf{a}|} T(s_i,a_i,s_{i-1}).\\ \end{aligned}$$

Essentially, this repeatedly convolves the elementary transition dynamics, as determined by $\mathbf{a}$. The same idea applies to the cost as well.

#### Construction 2

(Cost composition (Rossi & Shell, 2022)) For discount $\gamma \in (0,1)$ and elementary cost function $C_{{\mathrm{exc}}}: S\times A\rightarrow \mathbb {R}$, the corresponding macro execution cost is $\mathbf{C} _{{\mathrm{exc}}}^{\gamma }: S\times \mathbf{A}\rightarrow \mathbb {R}$, which we define as

$$\begin{aligned} \mathbf{C} _{{\mathrm{exc}}}^{\gamma }&\left( s,\mathbf{a}\right) = \mathbf{C} _{{\mathrm{exc}}}^{\gamma } \left( s,a_1 \!\ldots a_{|\mathbf{a}|}\right) \\&=\! \sum _{k=0}^{|\mathbf{a}|-1}\gamma ^{k} \hspace{-1.1ex} \sum _{\begin{array}{c} s_1 \!\ldots s_{k+1}\in S^{k+1}\\ \text {where } s_1 = s \end{array}} \hspace{-3.5ex} C_{{\mathrm{exc}}} (s_{k+1},a_{k+1}) \prod _{i=1}^{k} T(s_{i+1},a_i,s_i). \end{aligned}$$

Notice how $\gamma$ ensures costs incurred later, owing to sequences of actions, are diminished correctly.

For simplicity, we will treat state spaces where *S* is finite; also, as will be described below, macro-action sequences will be computed up to some bounded length.

### Solution concept for a mdpans

The task at hand, as with all planning, is to make immediate decisions with a view to future costs. In our case, one makes immediate decisions factoring in the future formation of future schedules (i.e., the cost represented by $C_{{\mathrm{sch}}}$). To help make this —admittedly opaque—statement clearer, we present first the Bellman-like recurrence for an mdpans, and the unpack the policy that such a function encodes implicitly.

#### Problem 1

Given any mdpans $$M=\langle $$$$ S, A, T, $$$$C_{{\mathrm{exc}}}, $$$$C_{{\mathrm{obs}}}, $$$$C_{{\mathrm{sch}}}, $$$$ \gamma , \xi ^0\rangle $$, compute the optimal state-value function $$\mathrm{U}: S $$$$ \times \mathbb {N}^*$$$$\rightarrow \mathbb {R}$$, which we define recursively as

1 $$\begin{aligned} \mathrm{U}(s,\varepsilon )&= C_{{\mathrm{sch}}} (s) + \hspace{-6pt}\min _{k_1\!\ldots k_\ell \in \mathbb {N}^+}\hspace{-4pt} \mathrm{U}(s,k_1k_2\!\ldots k_\ell ), \end{aligned}$$

2 $$\begin{aligned} \mathrm{U}(s,k_1k_2 \!\ldots k_\ell )&= C_{{\mathrm{obs}}} (s) + \min _{\mathbf{a} \in A^{k_1}} \Big [\mathbf{C} _{{\mathrm{exc}}}^{\gamma } (s, \mathbf{a})\; + \\&\hspace{0.2cm} \mathbf {\gamma }(s,\mathbf{a}) \sum _{s'\in S} \mathbf{T}(s', \mathbf{a}, s)\,\mathrm{U}(s', k_2 \!\ldots k_\ell )\Big ] \nonumber \\&\hspace{3.2cm}\text {with }\mathbf {\gamma }(s, \mathbf{a}) = \gamma ^{|\mathbf{a}|},\nonumber \end{aligned}$$

where the function $\mathrm{U}$’s second argument is a (potentially empty) sequence of positive time increments.

By construction, possessing $\mathrm{U}(\cdot ,\cdot )$ enables one to determine the actions the system ought to take. This is clearest operationally, sketched as a concrete sequence of steps. At initialization the system begins in some state $s^0\sim \xi ^0$. It may then obtain a schedule $\mathbf{k}^0 = k_1^0k_2^0\!\ldots k_\ell ^0 = \mathop {\mathrm {arg\,min}}\limits \mathrm{U}(s^0, \varepsilon )$. This details when states will be known in the near future, i.e., the number of elementary stages between check-ins. To act, the system obtains macro-action $\mathbf{a}^0_1 = \mathop {\mathrm {arg\,min}}\limits \mathrm{U}(s^0, \mathbf{k}^0)$. Since $|\mathbf{a}^0_1| = k_1^0$, execution of this macro-action brings the system precisely up to the point where the next state is disclosed. Say that state is $s'$, then the next action is obtained as $\mathbf{a}^0_2 = \mathop {\mathrm {arg\,min}}\limits \mathrm{U}(s', k_2^0\!\ldots k_\ell ^0)$. This repeats and once the time $k_1^0+ k_2^0 + \cdots + k_\ell ^0 = \sum _{j=1}^{\ell } k_j^0$ has elapsed, the next schedule will be obtained via $\mathop {\mathrm {arg\,min}}\limits \mathrm{U}(\cdot , \varepsilon )$. It is a function of the system’s state at that juncture.

Crucially, the value function associates a cost-to-go with states at precisely those moments when the system can actually have knowledge of the current state. (This fact is true with the standard mdp Bellman relation too, albeit a trivial consideration in that case). Schedules are obtained via $\mathfrak {s}:S \rightarrow \mathbb {N}^+$,

3 $$\begin{aligned} \mathfrak {s}(s) = \mathop {\mathrm {arg\,min}}\limits \mathrm{U}(s, \varepsilon ). \end{aligned}$$

The schedule’s purpose is twofold. It establishes when the next phase of the schedule will be determined, expressed as the sum of its comprising elements; and it declares when state information will be available in the interim.

The solution to Problem 1 may be understood as having both a ‘big-step’ progression from scheduling instant to scheduling instant, and ‘little-step’ behavior respecting the commitment to compartmentalized subsequences that have no state observations in between. Given a schedule $\mathbf{k}^t = k_1^tk_2^tk_3^t\!\ldots k_\ell ^t$, there are $\ell = |\mathbf{k}^t |$ macro-actions generated via

$$\begin{aligned} \mathop {\mathrm {arg\,min}}\limits \mathrm{U}&(s^t, k_1^tk_2^tk_3^tk_4^t\!\ldots k_\ell ^t),\\ \mathop {\mathrm {arg\,min}}\limits \mathrm{U}&(s^{t+|k_1^t|}, k_2^tk_3^tk_4^t\!\ldots k_\ell ^t),\\ \mathop {\mathrm {arg\,min}}\limits \mathrm{U}&(s^{t+|k_1^t|+|k_2^t|}, k_3^tk_4^t\!\ldots k_\ell ^t),\\ \mathop {\mathrm {arg\,min}}\limits \mathrm{U}&(s^{t+|k_1^t|+|k_2^t|+|k_3^t|}, k_4^t\!\ldots k_\ell ^t),\\&\qquad \vdots \\ \mathop {\mathrm {arg\,min}}\limits \mathrm{U}&(s^{t+|k_1^t|+|k_2^t|+\dots +|k_{\ell -1}^t|}, k_\ell ^t), \end{aligned}$$

where the states $$s^t, s^{t+|k_1^t|}, $$$$s^{t+|k_1^t|+|k_2^t|}, $$$$s^{t+|k_1^t|+|k_2^t|+|k_3^t|},$$$$\dots , $$$$s^{t+|k_1^t|+|k_2^t|+\dots +|k_{\ell -1}^t|}$$ carry lengthy superscripts to clearly indicate how fresh information, obtained since *t*, is used. Rather than a sequence, the actions are thus more faithfully visualized as a tree of depth $$\ell $$ that branches on states.

The scheduling cost $C_{{\mathrm{sch}}}$ appears in Equation (1), being incurred only once for each big step. The observation cost $C_{{\mathrm{obs}}}$ shows up in Equation (2), precisely once per macro-action, as an observation occurs at the beginning of each macro-action. The future expected values are discounted by the time until the next observation; the definition of $\mathbf {\gamma }(s,\mathbf{a})$ expresses this simply using the length of $\mathbf{a}$. When $C_{{\mathrm{obs}}} \le 0$, actions with $|\mathbf{a}| > 1$ offer no advantage over elementary ones; this also means optimal schedules ought to consist of $k_i$’s that equal 1. We will examine, in subsequent sections, how extremal choices of $C_{{\mathrm{sch}}}$ affect the optimal solutions to Problem 1. (The impatient reader may wish to examine Fig. 4 and the discussion of the special cases in the caption serving as a précis).

Equations (1)–(2) say that schedule negotiations only occur at time slots that are also state check-in times. A fair question to raise is whether one might benefit from the relaxation of this constraint. Suppose a solution is proposed that opts to decide on a schedule at a point in time $t_p$ between the check-ins at time $t_i$ and $t_{i+1}$, i.e., $t_i< t_p < t_{i+1}$. Observe that since no information is revealed between check-ins, the proposed approach can do no better than a solution shifted back to time $t_i$, which would then satisfy the form demanded by Equations (1)–(2). So, the answer is *no*.

Finally, the reader may argue that since $\mathbb {N}^+$ is not finite, the $(\arg )\min _{k_1\!\ldots k_\ell \in \mathbb {N}^+}$ above, technically, may not exist and ought to be an $(\arg )\inf _{k_1\!\ldots k_\ell \in \mathbb {N}^+}$. This will not concern us as, in computing solutions, we will bound the length of macro-actions via a parameter *m*. Such limits may arise naturally from the system’s constraints: the gliders in Example 1’s setting will have a maximum depth they can tolerate. (Also, we will return to explicitly consider the case when $\ell \rightarrow \infty$ in Sect. 4.3.1).

### Synchronized multi-robot systems: parallel composition

The definitions thus far model sparse observations subject to costs. We now give the final definitional piece of the problem needed in order to tackle the systems comprising the core of the paper: composition for the multiple robot case. For a system of *n* robots, with each $i \in \{1,\dots ,n\}$ having states $S_i$ and actions $A_i$, any specific sets $\mathcal {S} \subseteq \big \{{\boldsymbol{\langle }}{s^{{\!\!}^{{(\hspace{-0.4pt}1 \hspace{-0.4pt})}\!}} {\boldsymbol{|}}s^{{\!\!}^{{(\hspace{-0.4pt}2 \hspace{-0.4pt})}\!}} {\boldsymbol{|}}{\cdots }{\boldsymbol{|}}s^{{\!\!}^{{(\hspace{-0.4pt}n \hspace{-0.4pt})}\!}}}{\boldsymbol{\rangle }} :s^{{\!\!}^{{(\hspace{-0.4pt}i \hspace{-0.4pt})}\!}} \in S_i \big \}$ and $\mathcal {A} \subseteq \big \{{\boldsymbol{\langle }}{a^{{\!\!}^{{(\hspace{-0.4pt}1 \hspace{-0.4pt})}\!}} {\boldsymbol{|}}a^{{\!\!}^{{(\hspace{-0.4pt}2 \hspace{-0.4pt})}\!}} {\boldsymbol{|}}{\cdots }{\boldsymbol{|}}a^{{\!\!}^{{(\hspace{-0.4pt}n \hspace{-0.4pt})}\!}}}{\boldsymbol{\rangle }} :a^{{\!\!}^{{(\hspace{-0.4pt}i \hspace{-0.4pt})}\!}} \in A_i \big \}$ will be termed *joint states* and *joint actions*, respectively. The key definition is as follows.

#### Definition 2

An *actively synchronized multi-robot* mdp for a system of *n* robots is a mdpans $$M=\langle$$$$ \mathcal {S}, $$$$ \mathcal {A}, $$$$ \mathcal {T}, $$$$C_{{\mathrm{exc}}}, $$$$C_{{\mathrm{obs}}}, $$$$ C_{{\mathrm{sch}}}, $$$$\gamma , \xi ^0\rangle $$, defined over joint states and joint actions, i.e., it has states $${\boldsymbol{\langle }}{s^{{\!\!}^{{(\hspace{-0.4pt}1 \hspace{-0.4pt})}\!}} {\boldsymbol{|}}s^{{\!\!}^{{(\hspace{-0.4pt}2 \hspace{-0.4pt})}\!}} {\boldsymbol{|}}{\cdots }{\boldsymbol{|}}s^{{\!\!}^{{(\hspace{-0.4pt}n \hspace{-0.4pt})}\!}}}{\boldsymbol{\rangle }} \in \mathcal {S}$$ and actions $${\boldsymbol{\langle }}{a^{{\!\!}^{{(\hspace{-0.4pt}1 \hspace{-0.4pt})}\!}} {\boldsymbol{|}}a^{{\!\!}^{{(\hspace{-0.4pt}2 \hspace{-0.4pt})}\!}} {\boldsymbol{|}}{\cdots }{\boldsymbol{|}}a^{{\!\!}^{{(\hspace{-0.4pt}n \hspace{-0.4pt})}\!}}}{\boldsymbol{\rangle }} \in \mathcal {A}$$.

The transitions employed are for macro–joint actions built via Construction 1 on single time-step joint actions, i.e., the elements comprising $\mathcal {A}$. The transition function for those elements will typically model interactions, but if the robots have no couplings (e.g., they are fully independent/share no resources) it may be formed by directly composing the *n* individual actions. One point of optimization is that joint actions are permitted to be a subset of the possibly combinatorially large space of available choices.

For a concrete instance, return briefly to Example 1. If the pair of underwater gliders are denoted $\hspace{0.3pt}\ell \hspace{0.3pt}$ and $r$, with former in state $s^{{\!\!}^{{(\hspace{-0.4pt}\hspace{0.3pt}\ell \hspace{0.3pt}\hspace{-0.4pt})}\!}}$ and the latter in $s^{{\!\!}^{{(\hspace{-0.4pt}r \hspace{-0.4pt})}\!}}$, then the system’s state is ${\boldsymbol{\langle }}{s^{{\!\!}^{{(\hspace{-0.4pt}\hspace{0.3pt}\ell \hspace{0.3pt}\hspace{-0.4pt})}\!}} {\boldsymbol{|}}s^{{\!\!}^{{(\hspace{-0.4pt}r \hspace{-0.4pt})}\!}}}{\boldsymbol{\rangle }}$; joint actions will also, of course, involve two slots, the first for robot $\hspace{0.3pt}\ell \hspace{0.3pt}$, the second for robot $r$. Suppose that state is known to be ${\boldsymbol{\langle }}{s^{{\!\!}^{{(\hspace{-0.4pt}\hspace{0.3pt}\ell \hspace{0.3pt}\hspace{-0.4pt})}\!}} {\boldsymbol{|}}s^{{\!\!}^{{(\hspace{-0.4pt}r \hspace{-0.4pt})}\!}}}{\boldsymbol{\rangle }}$ because the pair generated a joint observation at time *t*, meaning that both gliders surfaced, collected gps data, and communicated their positions. Then, if they possess a copy of the optimal value function $\mathrm{U}$, the gliders (each) can determine the schedule $\mathbf{k}^{t} = \mathfrak {s}\big ({\boldsymbol{\langle }}{s^{{\!\!}^{{(\hspace{-0.4pt}\hspace{0.3pt}\ell \hspace{0.3pt}\hspace{-0.4pt})}\!}} {\boldsymbol{|}}s^{{\!\!}^{{(\hspace{-0.4pt}r \hspace{-0.4pt})}\!}}}{\boldsymbol{\rangle }}\big )$. They can also (each) determine the next joint macro-action:

$$\begin{aligned} \mathbf{a}^{t}&= \mathop {\mathrm {arg\,min}}\limits \mathrm{U}\big ({\boldsymbol{\langle }}{s^{{\!\!}^{{(\hspace{-0.4pt}\hspace{0.3pt}\ell \hspace{0.3pt}\hspace{-0.4pt})}\!}} {\boldsymbol{|}}s^{{\!\!}^{{(\hspace{-0.4pt}r \hspace{-0.4pt})}\!}}}{\boldsymbol{\rangle }}, \mathbf{k}^{t}\big )\\&= \big ({\boldsymbol{\langle }}{a^{{\!\!}^{{(\hspace{-0.4pt}\hspace{0.3pt}\ell \hspace{0.3pt}\hspace{-0.4pt})}\!}}_1 {\boldsymbol{|}}a^{{\!\!}^{{(\hspace{-0.4pt}r \hspace{-0.4pt})}\!}}_1}{\boldsymbol{\rangle }}, {\boldsymbol{\langle }}{a^{{\!\!}^{{(\hspace{-0.4pt}\hspace{0.3pt}\ell \hspace{0.3pt}\hspace{-0.4pt})}\!}}_2 {\boldsymbol{|}}a^{{\!\!}^{{(\hspace{-0.4pt}r \hspace{-0.4pt})}\!}}_2}{\boldsymbol{\rangle }}, \dots , {\boldsymbol{\langle }}{a^{{\!\!}^{{(\hspace{-0.4pt}\hspace{0.3pt}\ell \hspace{0.3pt}\hspace{-0.4pt})}\!}}_m {\boldsymbol{|}}a^{{\!\!}^{{(\hspace{-0.4pt}r \hspace{-0.4pt})}\!}}_m}{\boldsymbol{\rangle }}\big ), \text {where } m = {|\mathbf{a}^t|}. \end{aligned}$$

For actions to execute individually, they cleave the action sequence in two: glider $\hspace{0.3pt}\ell \hspace{0.3pt}$ will perform the sequence of actions $(a^{{\!\!}^{{(\hspace{-0.4pt}\hspace{0.3pt}\ell \hspace{0.3pt}\hspace{-0.4pt})}\!}}_1, a^{{\!\!}^{{(\hspace{-0.4pt}\hspace{0.3pt}\ell \hspace{0.3pt}\hspace{-0.4pt})}\!}}_2, \dots , a^{{\!\!}^{{(\hspace{-0.4pt}\hspace{0.3pt}\ell \hspace{0.3pt}\hspace{-0.4pt})}\!}}_m)$, while *r* enacts the sequence $(a^{{\!\!}^{{(\hspace{-0.4pt}r \hspace{-0.4pt})}\!}}_1, a^{{\!\!}^{{(\hspace{-0.4pt}r \hspace{-0.4pt})}\!}}_2, \dots , a^{{\!\!}^{{(\hspace{-0.4pt}r \hspace{-0.4pt})}\!}}_m)$. The gliders execute their individual sequence of actions without reference to the other robot for the times $t, t+1, t+2, \dots , t+m$. Thence, as they surface again, the process repeats. We will return to a final discussion of this example below, in Sect. 4.2.

We remark that this model is suitable for decentralized execution because the formulation, albeit not very explicitly, expresses a degree of synchrony in the robots’ activities. To be explicit, three specific assumptions are needed. Firstly, like all mdps, the observation (or check-in) reveals state perfectly—hence, multiple agents receive the same observation, namely, the state. Secondly, the agents, given the same state, will select the same action—in the decentralized case this requires a consistent tie-breaking mechanism. Third, when joint-actions are split into parts during decentralized execution, each agent’s execution of its part takes the same amount of time; the underlying stochastic process in a standard mdp progresses from stage to stage between actions, and the same is required of the system here. The topic of Sect. 6 is to relax these assumptions.

## Solutions to Markov decision process with actively negotiated schedules

When Dynamic Programming is employed for the recursion in Equations (1)–(2) it allows solutions to be built incrementally with the potential for different schedules to reuse some computation. Consider that, for all $s\in S$, both $\mathrm{U}(s,k_1k_2 \!\ldots k_\ell )$ and $\mathrm{U}(s,k'_1k_2 \!\ldots k_\ell )$ use values of $\mathrm{U}(\cdot ,k_2 \!\ldots k_\ell )$. And, with a further level of indirectness, $\mathrm{U}(s,k'_0k'_1k_2 \!\ldots k_\ell )$ depends on values of $\mathrm{U}(\cdot ,k_2 \!\ldots k_\ell )$ too; indeed, this is true for any schedule ending with $k_2 \!\ldots k_\ell$. Once a macro-action arrives at state *s*, it matters not how it got there (e.g., whether the macro-action was of length $k_1$ or $k'_1$, respectively)—the Markov property ensures that everything needed about the *past* is in state *s* itself. The essential information needed about the *future* is the sequence of intervening stages for the check-ins that are yet to come.

**Fig. 3:**
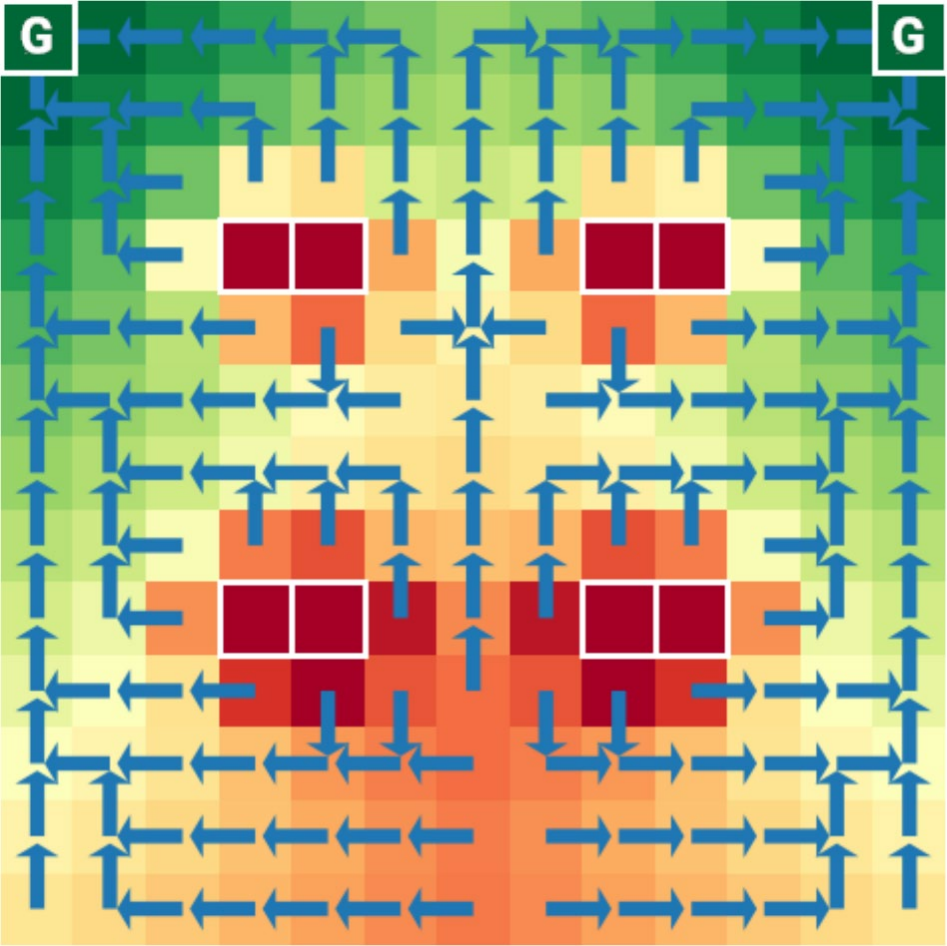
Navigation within a small grid. An agent with imprecise motions navigates in a simple rectangular world. The two locations marked with ’G’ in the upper left and right are goal cells; the eight red cells boxed in white as pairs make up four obstacles, and there is an outer boundary wall too. The agent has 5 actions: moving in each of the cardinal directions, and remaining stationary. The state value function is encoded as cell color (red being most costly, to green least), and the blue superimposed arrows show actions which minimize costs associated with each state

**Fig. 4:**
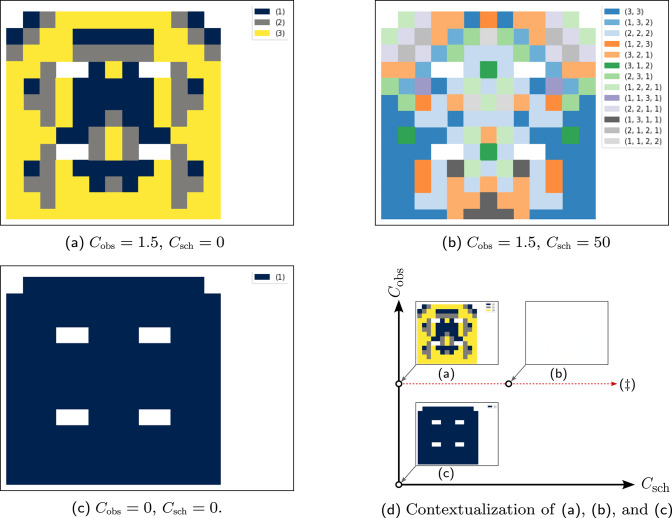
Concrete schedules resulting from differing cost values. The solution to an mdpans is a policy which encodes (i) sequences of actions to be taken directly, (ii) the immediate schedule describing a commitment to the occurrence of some future state observations, and (iii) when the next further portion of the schedule is to be determined. The visualization above shows optimal schedules for the navigation problem of Fig. 3, but with differing check-in ($C_{{\mathrm{obs}}}$) and scheduling ($C_{{\mathrm{sch}}}$) cost functions —the execution cost ($C_{{\mathrm{exc}}}$) corresponds to the description of the grid world provided in the text and remains fixed. Here, action and schedule sizes are limited with $N = 3$ and $T=6$. The plots of the policies elide detail of specific action choices, but use colors to illustrate both the state-varying and cost-dependent nature of optimal schedule $\mathfrak {s}(\cdot )$ obtained via Eq. (3). In the simplest case of **c**, we see standard mdp behavior because observations are obtained at every step. With check-in cost increased in **a**, macro-actions avoid paying the penalty by taking longer actions, seen in sequences with 2 steps (gray) or 3 steps (yellow). Increasing in $C_{{\mathrm{sch}}}$ in **b** means that, while **a** picks a new schedule after each check-in, multi-stage sequences of check-ins are produced (colors contrast to show diversity). Sub-figure **d** provides a rationale for the relative spatial placement of the plots. Subsequent sections will turn attention to two special cases. The case of **a**, where $C_{{\mathrm{sch}}} =0$, is discussed in Sect. 4.2. The red arrow showing increasing scheduling cost prefigures the case with $C_{{\mathrm{sch}}} \rightarrow \infty$, depicted as ($\ddagger$) , which is the subject of the discussion in Sect. 4.3

Nevertheless, computing function $\mathrm{U}$ requires searching over all possible schedules $k_1\!\ldots k_\ell$ of all possible lengths. In practical applications, one can limit the maximum time between check-ins to some *N*, so each $0 < k_i \le N$. As the number of such check-ins before establishing updated schedules itself can grow, this is a large space. For a bounded duration till the next scheduling step, *T*, there are exponentially many choices of $\mathbf{k} = k_1k_2 \!\ldots k_{|\mathbf{k}|}$ such that $\sum _{j=1}^{|\mathbf{k}|} k_j \le T$. This fact will motivate the study of two special cases, namely, the case where, for all states, the rescheduling cost $C_{{\mathrm{sch}}}$ is zero, and the case where it is infinite. These extreme points cover meaningful scenarios. First, however, we can get some insight into the general form of Problem 1 through the study of some small-scale simulation experiments.

### A small grid world problem

Consider an agent navigating within a simple grid world amidst obstacles. Figure 3 shows the 13$\times$13 environment with obstacles. The agent’s actions are noisy: when commanded to move to a neighboring cell in a given direction, the agent can drift into any of the undesired directions with 10 % probability each. Additionally, the agent has a driftless no-op action to remain stationary. Each of these actions has unit cost, collision with a wall has cost 50, collision with an obstacle 100, and arriving at the goal a cost of -250. The color of cells in Fig. 3 provides a visual encoding of the state value function (cost decreasing linearly from red to green), computed with the discount $\gamma = 0.95$. The blue arrows show the cost minimizing action associated with each state (i.e., the optimal policy) obtained by treating this as a standard single-step mdp.

We use exhaustive enumeration of schedule options and construction of a standard mdp. The idea is that one way to represent the recursion is to move the agreed-upon schedule for the next check-ins to the state: i.e., conceive of $\mathrm{U}(s, \mathbf{k})$, the optimal expected discounted cost from state *s* when the agent has agreed to subsequent observations at times $\mathbf{k}$ before the next schedule re-negotiation, as handling a cost associated with $(s, \mathbf{k})$ as a ‘state pair’. Figure 4 shows schedules obtained after computing $\mathrm{U}$. The mdp is then solved using standard tools (our implementation allows either value iteration or linear programming). The three schedules correspond to different regimes of behavior, with sub-figure d serving as a legend in helping to emphasize their relationships: y The first regime is a special case of the second, while the second is a special case of the third. The third case maintains meaningful distinctions between big- and little-step progression, while in the other two those collapse. In Figure 4a, there is information encoded in the length of macro-actions, while c this is no longer the case.

These examples illustrate that policies of the general form described in Sect. 3.3 can be successfully obtained algorithmically, and that they fit one’s intuition given the relative cost magnitudes involved.

Of course, searching the space of general schedules becomes prohibitive as *N* and *T* grow. This motivates the study of two special cases of Problem 1.

### A special case: no rescheduling cost

When the rescheduling cost is zero, agents can renegotiate the schedule at every check-in without any loss of generality. This simplification makes the problem significantly more tractable. In this case, we use $\mathrm {U_{{\!}_0}}$ to denote the optimal state value function. Formally, $\mathrm {U_{{\!}_0}}: S \rightarrow \mathbb {R}$ satisfies the following Bellman recurrence:

4 $$\begin{aligned} \mathrm {U_{{\!}_0}}(s)&= C_{{\mathrm{obs}}} (s) + \min _{\mathbf{a} \in A^{+}} \mathbf{C} _{{\mathrm{exc}}}^{\gamma } (s, \mathbf{a})\; \\&\quad + \mathbf {\gamma }(s,\mathbf{a}) \sum _{s'\in S} \mathbf{T}(s', \mathbf{a}, s)\,\mathrm {U_{{\!}_0}}(s').\nonumber \end{aligned}$$

There is no longer any need for function $\mathfrak {s}(\cdot )$ because $\mathbf{a}^t = \mathop {\mathrm {arg\,min}}\limits \mathrm {U_{{\!}_0}}(s^t)$ yields the macro-action to be executed, and the next check-in/synchronization time is encoded through the macro-action’s length—no explicit schedule or separate communication policy is required.

We remark that though this is a special case, the situation modeled with $C_{{\mathrm{sch}}} = 0$ is often quite natural. For instance, the gliders in Example 1 have a single logical activity that describes surfacing, obtaining a gps position, and broadcasting their pose. We can model this with $C_{{\mathrm{sch}}} = 0$ not because the cost of scheduling is subsumed by $C_{{\mathrm{obs}}}$ (which involves explicitly communicating anyway), but rather because complete information is available to all actors in the system after every check-in. Recall: a check-in always discloses global/joint state, but we are discussing *to whom* this is disclosed.

For the underwater gliders, there is never a need to fix a schedule beyond agreeing to the time of the next check-in. This is because, upon their next check-in, the pair will always again be in a situation with the state known to all, and hence they can readily determine the subsequent check-in point at that juncture. Indeed, any commitments other than the very next check-in will use information less fresh than is obtained by making the decision in a check-in per check-in, hand-to-mouth manner as done by the gliders. The gliders know the time until their upcoming check-in, know that they will then determine the subsequent one, and—applying this reasoning repeatedly—know this holds in perpetuity.

Though these statements appear to be about high-level knowledge, they are expressed in the recurrence above, albeit slightly hidden below the surface. Values of $\mathrm {U_{{\!}_0}}$ are associated with states, and the formulation constructed so that exact states are known when $\mathrm {U_{{\!}_0}}$ must be queried. And in Equation (4), function $\mathrm {U_{{\!}_0}}$ relates, recursively, to itself only at points where states would be known. The physical system will pass through several states in the real world; it skips from known state to known state, typically losing precise knowledge in between—but the value function $\mathrm {U_{{\!}_0}}$ is a relation only between these endpoints. Critically, choosing the size of these jumps (i.e., the time of the next observation) is part of what $\min _{\mathbf{a}\in A^{+}}$ optimizes. A Bellman relation for a traditional mdp expresses the fact that the optimal action minimizes total cost, computed as an immediate cost along with the requirement to behave optimally thereafter. It is recursive because there are subsequent points in time at which the agent faces essentially the same decision problem anew, with the Markov property permitting one to collapse a longer history and eliminate it from consideration. These interpretations also hold for $\mathrm {U_{{\!}_0}}$ and Equation (4). The statement above ‘holds in perpetuity’ denotes the fact that the recurrence in Equation (4) can be unwound arbitrarily deeply, with $\min _{\mathbf{a}\in A^{+}}$ operations at each level, and every subsequent portion taking a form identical to previous ones.

The glider case also emphasizes how our development uses two forms of composition: sequential, i.e., across time using the macro-action definitions from Sect. 3.2, and parallel, i.e., across robots using the joint actions and states from Sect. 3.4. As each glider uses gps at the surface, it obtains its own location, sensing a sort of ‘half state’; when followed by communication, this becomes the joint state, known by all. For each agent to act appropriately, there is no need for subsequent communication in determining their actions, so long as each agent has the state value function. Strictly, each agent can get away with less than the full value function, $\mathrm {U_{{\!}_0}}$, because it only needs to extract a ‘half action’ it can store this projection of $\mathop {\mathrm {arg\,min}}\limits \mathrm {U_{{\!}_0}}(s)$ for each *s*. (This assumes that they have the same $\mathrm {U_{{\!}_0}}$, obtained at planning time, and they break ties consistently). Definition 2 does not specify how joint states are obtained nor how macro–joint actions are transformed into actions for individual robots; in situations other than Example 1, decentralized execution may forgo all communication altogether. Sect. 5 will describe a formation-keeping demonstration with robot hardware where local sensing provides the requisite state information. We defer discussion of computational and experimental results for the $C_{{\mathrm{sch}}} = 0$ case until that point.

### A special case: infinite rescheduling costs

**Fig. 5:**
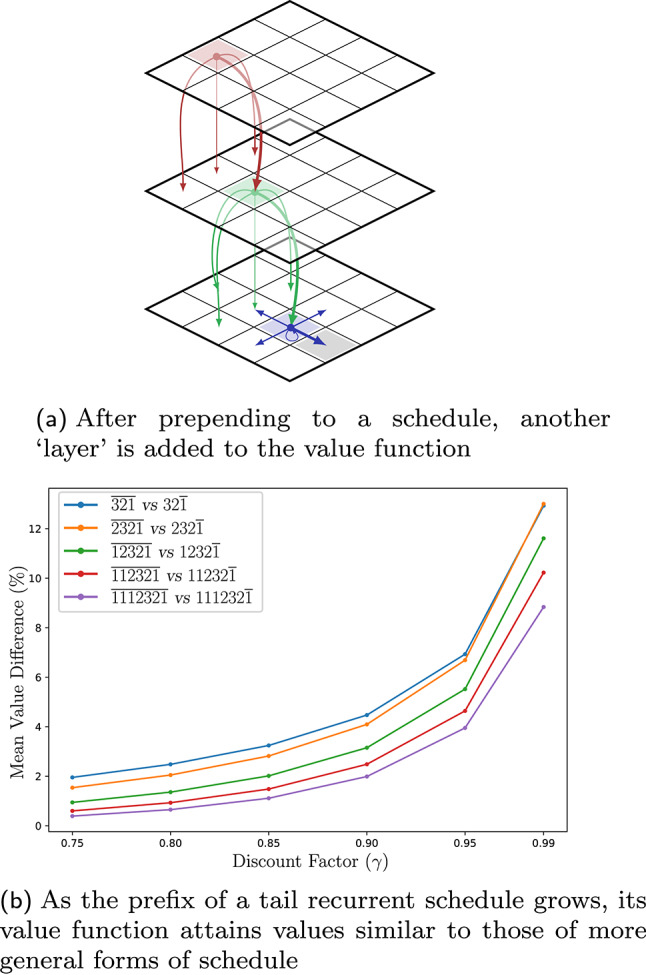
Schedules can be extended incrementally. A prepending operation, visualized as the addition of another ‘layer’ in **a**, allows new values to be computed using the existing ones. The plot in **b** quantifies how the tail’s contribution is diluted by greater discounting (which correspond to smaller values of $\gamma$)

We now turn to the opposite extreme from Sect. 4.2. As the value of $C_{{\mathrm{sch}}}$ is increased, the system is incentivized to avoid generating additional schedules. For sufficiently large $C_{{\mathrm{sch}}}$, the mdpans problem becomes one of finding a single schedule of check-ins that is effective across all the problem’s possible realizations. The agent or agents using the schedule are planning under uncertainty with observations that are intermittent, but have timing known via the pre-declared schedule. The big-step progression becomes, essentially, a single giant step, and all little steps are decisions about sequences of actions fitting a single schedule. Note that since Equation (1) describes the initial conditions, the system always pays the cost $C_{{\mathrm{sch}}}$ at least once. So when we speak of passing to the ‘limit’, this is really considering the avoidance of *re*-scheduling.

When might such a setting apply in practice? Consider Example 2, involving the uav/ugv team. The scenario as described has check-ins when the uav flies over the region. At those points, though the ugv learns its pose, the uav remains unaware of the ugv ’s location. For rescheduling, it is sufficient to have both ugv and uav know the joint state, which is feasible if the ugv can send information back to the uav. With broadcast from the uav to the ugv, and now information returned in the reverse direction, we term this the *full-duplex setting*. The machinery exemplified in Sect. 4.1 can be used for the full duplex setting.

But suppose, instead, we wish the ugv to avoid active data transmission, e.g., to improve its stealthiness, or save power, or to simplify its design. We then have a *half-duplex setting*, and one which is suitably modeled as $C_{{\mathrm{sch}}} \rightarrow \infty$. Notice that in this case, with a pre-declared schedule, the action-executing element of the system is just the ugv, and it obtains the state information it needs in the check-ins. The uav ’s task is simply to stick to the committed flyover schedule.

To formalize the problem, we will use $\mathrm {U_{{\!}_\infty }}$ to denote the optimal state value function, $\mathrm {U_{{\!}_\infty }}: S \times \mathbb {N}^\omega \rightarrow \mathbb {R}$ satisfying the following recurrence:

5 $$\begin{aligned} \mathrm {U_{{\!}_\infty }}(s,k_1k_2k_3k_4 \!\ldots ) & = C_{{\mathrm{obs}}} (s) + \min _{\mathbf{a} \in A^{k_1}} \mathbf{C} _{{\mathrm{exc}}}^{\gamma } (s, \mathbf{a})\; \\&\quad + \mathbf {\gamma }(s,\mathbf{a}) \sum _{s'\in S} \mathbf{T}(s', \mathbf{a}, s)\, \\ & \qquad \mathrm {U_{{\!}_\infty }}(s', k_2k_3k_4 \!\ldots ).\nonumber \end{aligned}$$

This is just Equation (2), where we have the technical generalization in that its second argument is no longer $\mathbb {N}^*$ but $\mathbb {N}^\omega$, the set of one-sided infinite sequences of positive integers. We term such a sequence an infinite schedule. For a given infinite schedule, Equation (5) allows one to produce the sequence of macro-actions to execute.

Finding the optimal infinite schedule requires searching over $\mathbb {N}^\omega$. To help tame this prospect, observe that if $\mathrm {U_{{\!}_\infty }}(\cdot , k_2k_3k_4\cdots )$, appearing on the right of Equation (5), is known then one merely needs to prepend $k_1$ to obtain the value on the left-hand side. Figure 5a provides a visualization of function  conceptually, showing the incremental process by which a blue schedule is extended, prepending a green step, which is then itself extended with a red step. Such schedule extension provides a way to add any finite prefix, but it requires an initial infinite schedule. Since the execution is discounted by $\gamma$, the effect of the tail diminishes as prefixes are prepended, and so it is sensible to use a starting point that is computationally convenient. We focus on schedules that are *tail recurrent*, by which we mean $\exists N_0 \in \mathbb {N}$ such that $k_{n+1} = k_n$, $\forall n \ge N_0$. For instance, $11123211111\!\ldots = 111232\overline{1}$ is tail recurrent. One advantage of a tail recurrent schedule is that we can compute a value function with only a single copy of the state space (so in Fig. 5a the bottom layer, in blue, would be such a schedule). An extension step requires a copy, but once the value has been obtained on those states, the original value function can be discarded. Prepending to a tail recurrent schedule gives another tail recurrent schedule, and the space complexity does not grow with schedule length.

As evidence for the claim regarding discounting, Figure 5b provides data showing how the tail’s impact decreases as more elements are prepended. The plot shows the difference in value function for a robot moving in a grid world problem with imperfect movement, akin to the problem instances in Sect. 4.1. The role the discount plays is as intuition would suggest: the difference takes longer to wash out with larger $\gamma$. For values of the discount factor below 0.99, the value difference between schedules with the same prefix, but different tail recurrences, is well below 10%; this suggests that the proposed selection of a tail-recurrent schedule, while suboptimal in general, is unlikely to have a large impact on the quality of the resulting solution. Further corroborating this observation is the fact (not shown in the figure) that, despite the differences in the value of function $\mathrm {U_{{\!}_\infty }}$, all actions (i.e., policies obtained via ‘$\mathop {\mathrm {arg\,min}}\limits$-ing’) were identical in all cases.

**Fig. 6:**
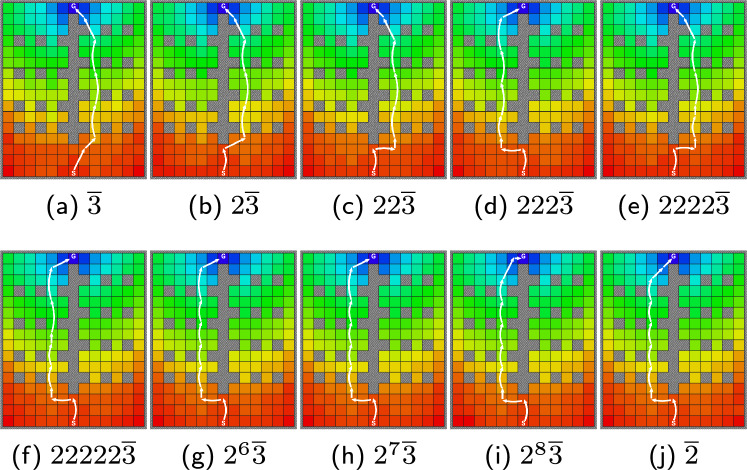
Incremental schedules changes can yield qualitative alterations in behavior. A navigation problem (similar to Fig. 3), with a complex arrangement of obstacles shown in gray, a start position denoted ‘S’ at the grid bottom and a single goal ‘G’ at the top; colors represent increasing state values, linearly from red to blue. In this scenario, when schedules are extended the optimal policies may switch between going left and right. In this scenario, when schedules are extended the optimal policies may switch between going left and right. The white arrows show the actions selected, following the trajectory corresponding to the maximum likelihood execution. Some arrow lengths are shorter than expected because the action involves a no-op action that does not modify the robot’s displacement

The approach described is sufficient to investigate small scale problems. Next, we present data that illustrate how schedule extension can result in non-trivial (even drastic, qualitative) changes to the policy of a schedule, with the grid world in Fig. 6. The world is constructed such that a $\overline{2}$ schedule would favor the west side due to the cadence 2 rows, while $\overline{3}$ would favor the east. Due to the center column, the agent cannot switch once it takes a side. At the start state, the choice is clear: go left if the stride is 2, go right if the stride is 3. As we start prepending $k=2$ check-ins to the $\overline{3}$ schedule, however, we notice that the policy flips directions for certain schedules, with $222\overline{3}$ going leftwards instead. This occurs since the prefix of the schedule, 222, brings the agent to states on the left that are superior for the remainder of the schedule, $\overline{3}$, than if it had gone right. As we continue prepending more $k=2$ check-ins, the flipping continues as the tail gets pushed farther north from the start state. The white curve in Fig. 6 consists of arrows to denote the highest probability outcome of the macro-actions (for each, its stride matches the schedule’s entry but, critically, actions can include no-ops warranted by the uncertainty).

Before reporting results related to the search for a schedule, we (briefly) introduce a further elaboration to the model that both (i) makes particular sense in the context of situations like the uav/ugv system, and (ii) enriches the notion of an optimal schedule.

#### A multi-objective view

**Fig. 7:**
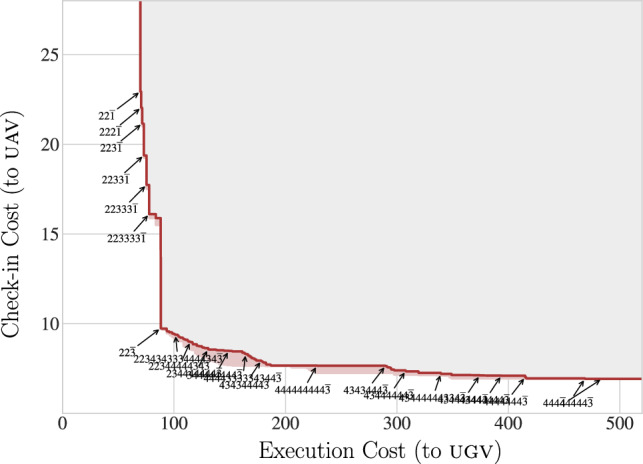
A Pareto frontier of schedules and navigation policies. Schedule and policy pairs can be plotted as a point in the plane above by computing two costs: the first reflecting the number of check-ins and the second the execution cost. The figure helps visualize how schedules involve an inherent trade-off: frequent observations provide more information but that may increase the fuel cost the navigating party is forced to bear. More desirable pairs appear toward the lower-left. The shaded gray area shows ‘dominated’ pairs, where one of the points marked with an arrow would either provide better check-in or better execution cost, or both. The Pareto frontier, characterizing non-dominated performance, is approximated by the red line of currently non-dominated solutions which have been found. The results reflect computation of prefixes up to length 15, extended incrementally from tail recurrent schedules; the light pink appearing below the red represents uncertainty still to be resolved, i.e., places where a not-yet found, improved policy may still lurk

The half-duplex setting results in a pre-determined schedule that the uav will fly; the ugv essentially plans subject to this commitment. Suppose we consider these more seriously as two different parties: we see that, for example, one bears the expense of the flight and the other the cost of terrestrial navigation. A schedule like $1111\!\ldots = \overline{1}$ (i.e., a standard mdp) would provide the ugv with maximal information, information that it can exploit to reduce the cost of its journey. The schedule $\overline{1}$ involves the uav performing a great many flights. Instead, if the ugv must cope with fewer check-ins, its actions must hedge to handle uncertainty, incurring greater cost. This tension in the consumption of the two types of fuel may matter if each party bears the respective cost.

Generalizing from single- to multi-objective optimization affords a way to pose problems involving a trade-off. For different schedules, consider the cost as a $2\times 1$-vector quantity, with the uav and the ugv ’s costs. Calling the former the ‘check-in cost’ and the latter the ‘execution cost’, different schedules can plotted in 2-dimensional space and a Pareto frontier of the non-dominated schedules identified. (In the multi-objective setting, an element is dominated if some other element has lower or equal cost on every dimension and strictly lower on some dimension). The idea is that appropriate compromises can be struck when informed by the Pareto frontier in the space of execution and observation costs. The frontier is a generalization of single-objective cases in the sense that the typical means of combining costs (e.g., summing because they come from a single budget) becomes a directly identifiable point on the front.

With a schedule $\mathbf{k} \in \mathbb {N}^\omega$, for the ugv, the execution cost is naturally described in $\mathbb {E}(\mathrm {U_{{\!}_\infty }}(s^0, \mathbf{k}))$ with the expectation taken over initial states, $s^0\sim \xi ^0$. There are many possible choices for the uav ’s check-in cost. Our model is to: mark a goal state (e.g., see the ‘G’ in Fig. 6) and compute the expected number of check-ins to arrive at the goal. This is both meaningful in navigation problems, as it gives a sense of how many flyovers would be expected, and can be conveniently computed by using the same infrastructure employed to compute policy values via a ‘counting cost’—a function that is 0 or 1 depending on whether the action is in the goal or not. We considered a $5\times 27$ grid world with obstacles appearing in twos and threes, essentially like the gray squares in Fig. 6. A Pareto frontier of schedules was computed and is visualized in Fig. 7 (in doing so, we made use of pruning optimizations; those details have been omitted, but we refer the interested reader to Zhong et al. (2023)). Notice how schedules with sparser check-ins (like $4444\overline{3}$) save check-in cost, but greatly increase the execution cost. The diagram shows that $22\overline{3}$ is a compromise which could be rather effective for both parties.

### Complexity analysis

The focus of this article is in formulating planning problem in which not only are actions selected, but commitments are made about how long until the next state observation will occur. And, in the general case when $C_{{\mathrm{sch}}} (s) \ne 0$, the problem determines the next time to settle the commitment of future check-in schedules. As mentioned at the end of Sect. 2 there remain opportunities to see if the approximation techniques developed for the specific case of $C_{{\mathrm{sch}}} (s) = 0$ can be generalized. Here, we will give an account of the size of solving an mdpans (i.e., computing the solution to Problem 1) by employing a standard mdp solver.

Let $\mathbb {A}(s,a)$ represent the cost of solving an mdp with *s* states and actions *a*, i.e., $s = |S|$ and $a = |A|$. For instance, using value iteration, for *m* iterations (typically $m \approx -\log \left( {1-\gamma }\right)$), then $\mathbb {A}(s,a) = O(m\cdot s^2 \cdot a)$, with no extra problem structure assumed (LaValle, 2006).        

Suppose that in searching for solutions, we consider the maximum time to reschedule is *T*, so that $k_1 + k_2 + \!\ldots + k_\ell \le T$. Then the $\min$ in Equation (1) searches over integer partitions. The ways to do this for exactly *T* is ${{2T-1}\atopwithdelims (){T-1}}$, where we pick $T-1$ separating markers from $2T-1$ slots, and ${{2T-1}\atopwithdelims (){T-1}} \le \frac{1}{\sqrt{2T}}2^{2T} \in O(4^{T})$. Which implies that the ways to do this for all $m \le T$ is $O(4^T)$. In total, the optimal-state value function will then have $O(4^T|S|)$ entries. Solving Equation (2) is a matter of thinking of those extra choices as encoded within states (see mention of this in Sect. 4.1). So, in order to obtain a complete expression we must determine the size of the action set: in general the largest actions we can have would be of length *T*, and hence there are $|A|^T$ such actions. Computation of Constructions 1 and 2, even very naïvely, requires at most $O(|S|\cdot T)$ and $O(|S|\cdot T^2)$. The cost would be order of $\mathbb {A}(4^T|S|, |A|^T)$, which for value iteration becomes $O(m\cdot |S|^2 (8|A|)^T)$.

 For practical guidance, as the next section details, $C_{{\mathrm{sch}}} (s) = 0$ problems with N = 3 take on the order of a minute on standard hardware, using our python implementation—though the total time is dominated by forming the mdp rather than solving it $N=3$.

## Case study and hardware demonstration

Next, we describe a formation-keeping problem that permitted investigation of the theory presented above in operation on the hardware we have available. Formation-keeping connects with and bears similarity to Example 1, sharing commonalities such as the need for agents to mutually decide when to make the next check-in; agents each also bear the responsibility of providing ‘half’ of the state information at those observation points. An interesting difference is that they perform a (sometimes fallible) action to make such observations, which, owing to mismatching measurements, occasionally turn out to be imperfect. (The effect of such imperfections will be investigated in Sect. 6).

**Fig. 8:**
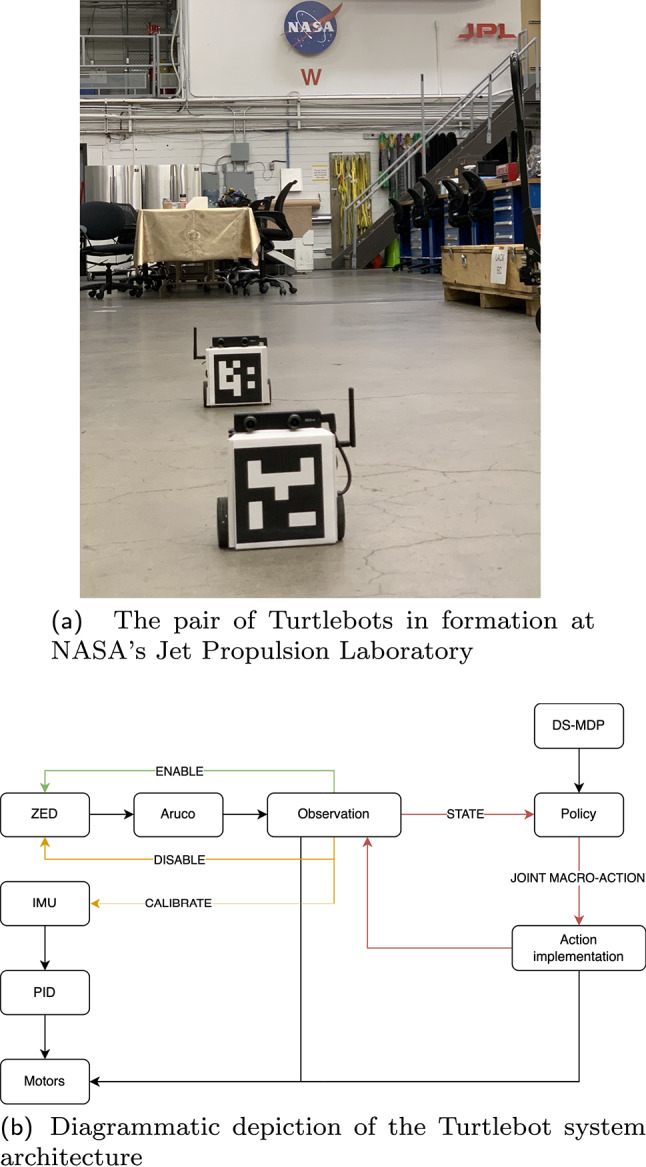
Robot system. A demonstration of a problem in which rescheduling occurs between a pair of Turtlebots which must stop and turn to discover their relative pose offsets via visual markers. A representation of the system in operation appears in Fig. 12

**Fig. 9:**
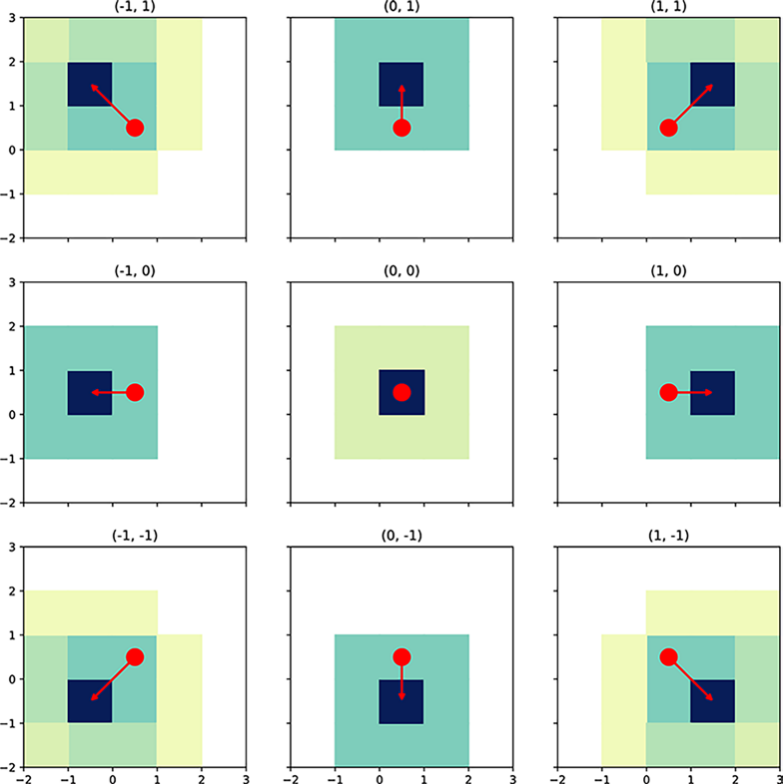
Formation-keeping transitions. Joint actions can be visualized as the difference in fine-grain adjustment of cross- and along-track positions, which we write as a pair $(\delta _x, \delta _\ell )$, with $\delta _x, \delta _\ell \in \{-1,0,-1\}$. The dynamics for the joint actions reflect that the fact that transitions are noisy (the shading gives a qualitative sense of the distribution of the outcome)

### Formation-keeping whilst driving problem

A pair of mobile robots are tasked with moving, indefinitely, in some general direction while keeping a predefined displacement between them. The robots navigate independently, making observations of their relative poses only sporadically during check-ins. The problem is symmetric and both robots become aware of the joint state after a check-in, fitting the case with $C_{{\mathrm{sch}}} = 0$ as discussed in Sect. 4.2. We model the problem by treating the joint state to be a description of the pose of one robot relative to the other, having each of the robots’ actions comprise fine-grained adjustment over a coarse baseline movement in the direction of motion. For this model, the state consists of two parameters: along-track error and cross-track error. A robot’s along-track distance to its partner is the separation along the axis of motion, and its cross-track distance is the separation along the perpendicular axis. For the formation moving east, positive along-track is east and positive cross-track is north. If the proper formation is that of the two robots being side by side, along-track separation would be zero with some desired cross-track separation when in formation. The cross-track and along-track *error* therefore measure how far off the current separations are from the desired formation separations.

Unlike a naïve mdp formulation with *x*/*y* coordinates for each robot, there are a number of advantages of this state description. Firstly, there is a reduction in the number of dimensions. Also, the state space need only accommodate the maximum separation of the robots, not the overall distance traveled (recall that they have a maintenance goal rather than an achievement one). The measurements do not require any global metrical reference frame, but merely measure distance along- and cross-track errors. Finally, each robot carries out its portion of the joint action independently of the other, but the joint actions have direct interpretations *in toto*: the pair get closer, separate, or maintain distance along each axis. Because some combinations of individual actions lead to nonsensical or redundant joint actions, those are omitted from the set of joint actions. Figure 9 helps visualize joint actions and their uncertainty.

**Fig. 10:**
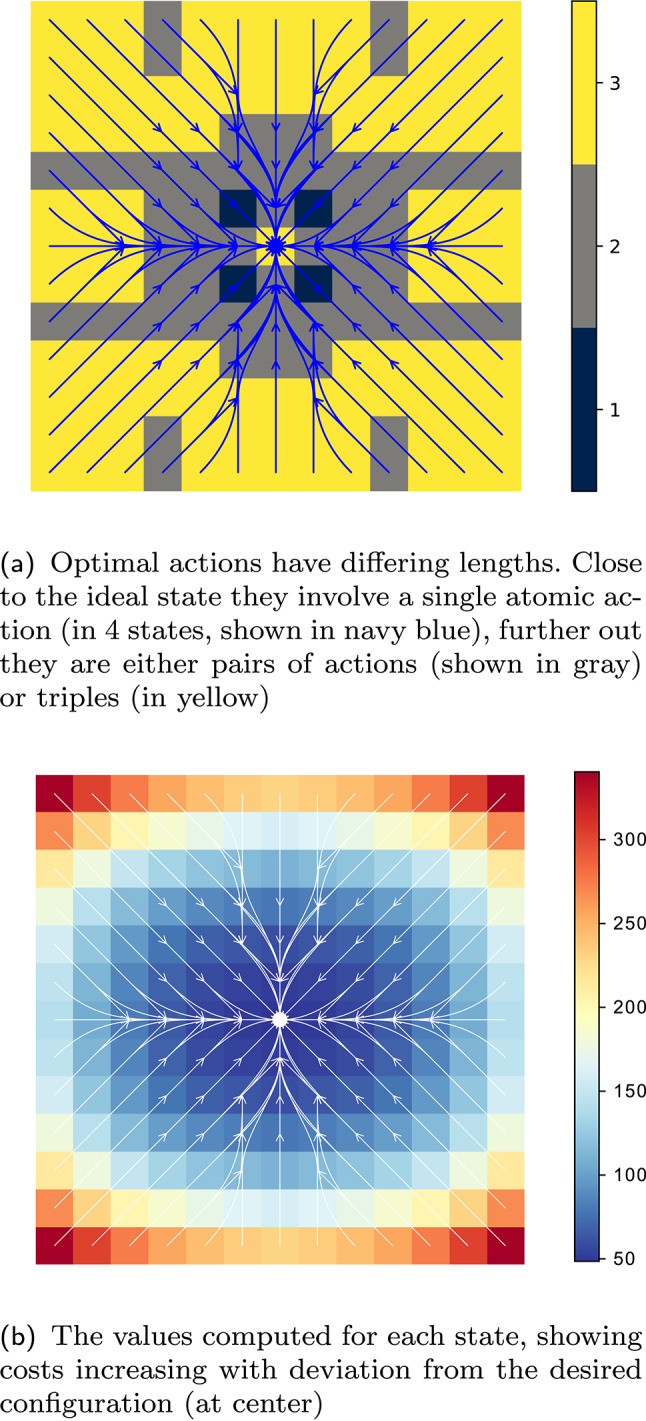
Formation-keeping policy. The policy for the formation-keeping problem with $N=3$, color-coded with length of each policy macro-action in **a** and the state values in **b**. The superimposed arrows (blue/white) show the sequences of optimal actions, giving a general visual sense of how actions reduce formation alignment error

**Fig. 11:**
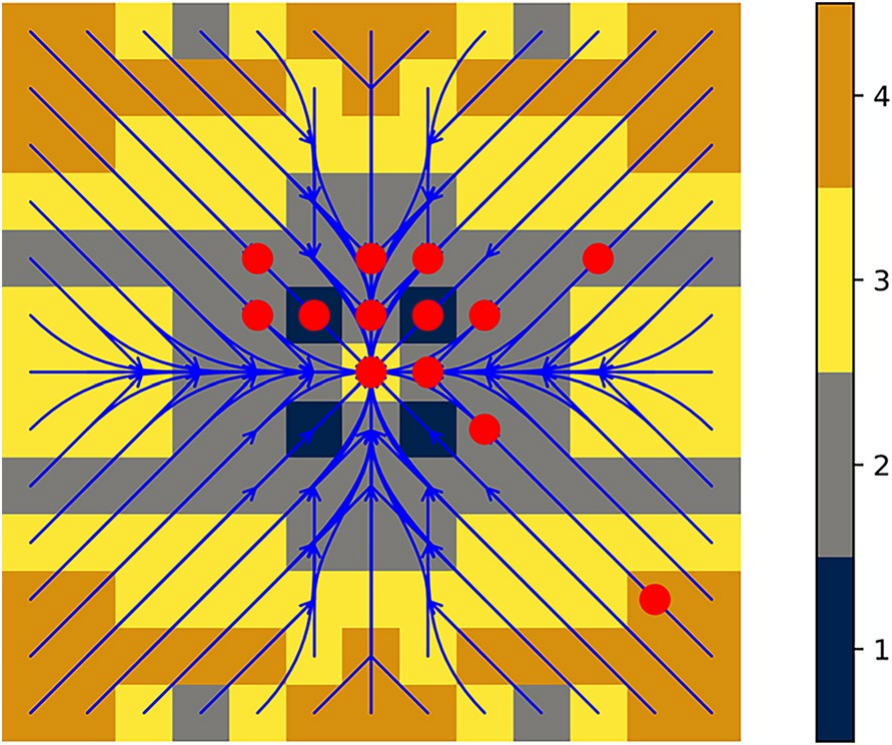
The optimal policy, analogous to Fig. 10a, now computed with $N=4$. Blue superimposed arrows show the sequences of optimal actions, and the legend on the right shows how colors encode sequence lengths. The overlaid red dots are states visited during the execution of the policy computed with $N=3$. The state marked with the red dot on the bottom-right was the (deliberately aberrant) initial starting position

**Fig. 12:**
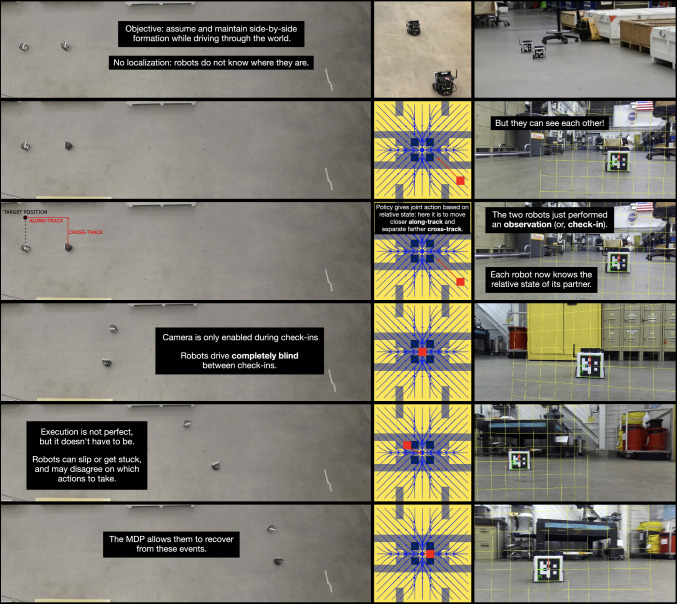
Hardware demonstration. Snapshots of the initial configuration, and a sequence of five check-ins, with an overhead view on the left, current policy state in the middle, and camera footage from upper robot on the right. Note that the yellow mesh superimposed on the video frames in the right column are reference frames centered on the ArUco tag; these lead, ultimately, to the computation of the red cell in the center column. The navy=1/gray=2/yellow=3 visualization of action sequence lengths maintains the convention of Fig. 10a. Video visible at https://youtu.be/Mv4Kgc9-1XY. (An inconsistency in the transition dynamics leads to a visual difference in the policy shown versus Fig. 10—still, both policies agree for every state visited, all actions executed)

### Hardware implementation

We assembled two Turtlebot3 Burger robots (Robotis, 2022) each with a single-board computer, an IMU, and a ZED Mini camera used to detect ArUco markers (Garrido-Jurado et al., 2014), see Fig. 8. The system has no global localization and each robot is only able to determine the relative spacing in the formation by directly sensing the other robot. The Turtlebots drive in arcs and cannot see each other when driving as the ArUco markers and cameras are positioned on the front of each robot. To obtain a check-in when the state is needed, the Turtlebots perform an expensive panning maneuver to seek each other out: the robots rotate toward and sweep the area where they believe their partner would be. With known marker size, camera parameters, and robot heading (obtained from the IMU), each robot can compute the location and orientation of the opposing ArUco marker and therefore derive both the cross- and along-track distances to its partner. After checking in, the robots return to their initial heading and drive according to the policy—reducing cross-track separation requires the robots to drive in arcs towards each other, while reducing along-track separation requires the lagging robot to drive at a higher velocity than the lead. Both robots are equipped with identical policies, from which they extract and execute their respective individual macro-actions.

For our setup, the check-in mechanism aided in the practical realization of the system. Disabling camera and ArUco processing except during observations yields far smoother motion as more CPU is given to our PID controller. While the IMU provides a fairly accurate heading, it drifts over time; it is used only during the check-in, and re-calibrated when the robot is stationary while the camera is releasing its resources. Thus, as our robots cannot drive well while scanning for markers, and cannot re-calibrate the gyroscope while moving—the check-in serves simultaneously for observation and for calibration.

### Results

In the context of the formation-keeping problem, the following discusses a specific generated policy as well as the effects of observation cost and transition uncertainty on other policies.

#### Computing policies

The mdp was solved using linear programming (LP), as a faster equivalent to value iteration. The majority of computation time was in transition composition, as well as building LP constraints. We use *N* to denote the bound of macro-action length. With $N=3$, the mdp took 21 s to build, the constraints took 27 s, and solving the LP took 6.6 s, for a total of 57 s. Solving the LP was thus only 11 % of the runtime.

**Fig. 13:**
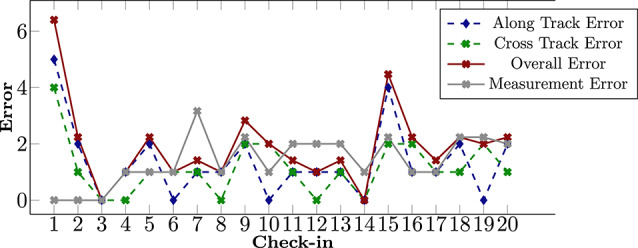
Hardware demonstration: errors. Cross-track, along-track, and overall (Euclidian distance) error with respect to the nominal formation measured by the robots. The policy is able to effectively drive the along-track and cross-track error close to zero. The plot also reports the measurement disagreement between the robots, i.e., the distance between the inter-rover offset measured by the first Turtlebot, and the inter-rover offset measured by the second Turtlebot. Even in presence of significant measurement disagreement, the policy is able to maintain the rovers close to the desired formation

Figure 10 displays the main policy we will examine in the following discussion of our experiments; it was computed using $N=3$. Colors indicate for each state the length of the macro-action dictated by the policy, allowing for visually identifying the number of steps until the subsequent check-in at a glance.

Figure 11 shows the policy for the larger bound of $N=4$, where the mdp, constraints, and LP took 6 min, 8.8 min, and 4.3 min, respectively, for a total of 20.7 min (of which solving was 20 %). The length-4 strides (orange) are only ever used around the boundary of the world, and indeed the execution of the $N=3$ policy never travelled to any of the regions which would have a length-4 stride in the $N=4$ policy—excepting the start state, which had the robots intentionally far apart to stress the experiment. As a result, there was insufficient benefit in raising *N* to 4 to justify its extra computation time, so we remained with $N=3$. (For direct visual confirmation, Figure 11 shows, as red dots, the states queried during the execution of Fig. 12).

#### Hardware execution

Figure 12 shows check-ins of the robots during execution, and Figure 13 shows the cross-track and along-track error. The onboard camera footage shows the ArUco marker of the other robot being recognized as the two robots look at each other during the check-in. In the first check-in, there is positive along-track error (upper robot is too far forward) and negative cross-track error (upper robot is too close), which translates to the red state in the policy diagram. The policy dictates for the robots to drive apart (upper goes further up, lower goes further down) and for the lower robot to drive at a higher velocity. This results in the new positions at the next check-in, one which is almost in formation. The robots believe they are in formation, as evidenced by the red state in the policy being in center, a result of the rounding error from discretizing the continuous world into grid cells. The action from that in-formation state is then for both robots to stay the course and move in sync.

#### Numerical experiments

We further explore the performance of the proposed policy through numerical simulations that compare its performance to mdp policies of fixed action length. The Turtlebots’s dynamics are captured through a unicycle model. The simulation captures execution errors in the commanded speed and heading, and measurement errors in the inter-rover distance; the variance of the errors (modeled as Gaussians) is qualitatively calibrated to the hardware experiments in Fig. 13. The same policy and speed controller deployed on hardware are used in the simulations. We compare the performance of the proposed policy with three policies with fixed strides between check-ins of one, two, and three steps; the one-step policy is represents a mdp solution to the problem. The starting location of the rovers is randomized.

**Fig. 14:**
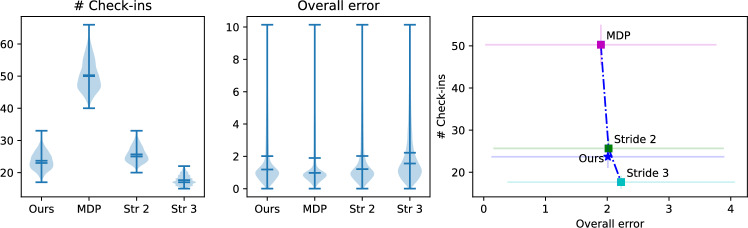
Numerical experiments: errors. Along-track, cross-track, and overall error, and number of check-ins for the proposed policy compared to fixed-stride policies with actions of length 1, 2, and 3. As the stride increases, error also increases and the number of check-ins decreases. The proposed policy is able to change the action length based on the state error, resulting in a lower number of check-ins while keeping error well-controlled. Exploiting this additional degree of freedom, it achieves performance that Pareto-dominates the stride-2 policy

**Fig. 15:**
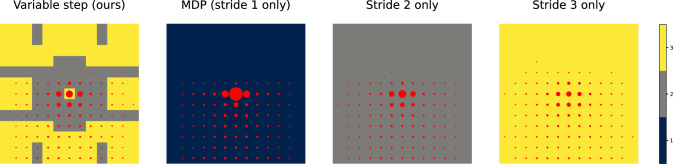
Numerical experiments: state distribution at check-in times. The color of cells denotes the length of the optimal action in that cell, as in Fig. 10a. Red dots denote the measured state when check-ins occur; the size of the red marker is proportional to the number of visits to a given state. The proposed policy is able to take longer steps when higher uncertainty can be tolerated, and shorter steps when precision is required, resulting in superior performance

The error and number of check-ins for 1000 Monte Carlo simulations are shown in Fig. 14; Figure 15 shows the measured state at the check-ins.

We observe that the proposed approach achieves performance intermediate between the stride-1 and stride-2 policies in terms of overall trajectory error, and between the stride-2 and stride-3 policies in terms of number of check-ins. The right-hand portion of Fig. 14 shows that our approach Pareto-dominates the stride-2 approach in expectation: the policy exploits the additional degree of freedom provided by taking longer steps when when higher uncertainty can be tolerated, reducing the number of check-ins with no negative effect (and, indeed, a very slight improvement) in formation tracking performance. Figure 15 sheds further light on this increase in performance by showing the state where the check-ins are obtained and the resulting policy length. The distribution of check-in states obtained by our policy is very similar to the stride-2 distribution; however, the proposed approach is able to take longer strides when the error is zero, decreasing the number of required check-ins, and takes precise, one-step strides when the error is close to zero, increasing tracking performance.

#### Properties of the policies

An aspect readily apparent in the policy is the dependence on distance—the farther the robots deviate from the formation, the longer the actions to take. Intuitively, an agent far from the target state should move as far as it can, since the optimal action will not change even if its trajectory slips marginally, whereas when closer it is more prone to overshooting into a different policy zone. For instance, an agent starting in a corner needs to move diagonally—shorter steps cause it to pass through states themselves requiring the same diagonal motion, so longer actions forgo the cost of check-ins which, in this case, add little extra value. Also, at the center, the policy is to execute long actions again, taking advantage of being in formation to perform a long stride.

Note also how not all policy macro-actions are to go straight, or diagonal. Some macro-actions are curved, being made up of different actions—for example, going diagonal then straight—and therefore are time-inhomogeneous (cf. Huang and Zhu (2021); Reisinger and Tam (2024)).

**Fig. 16:**
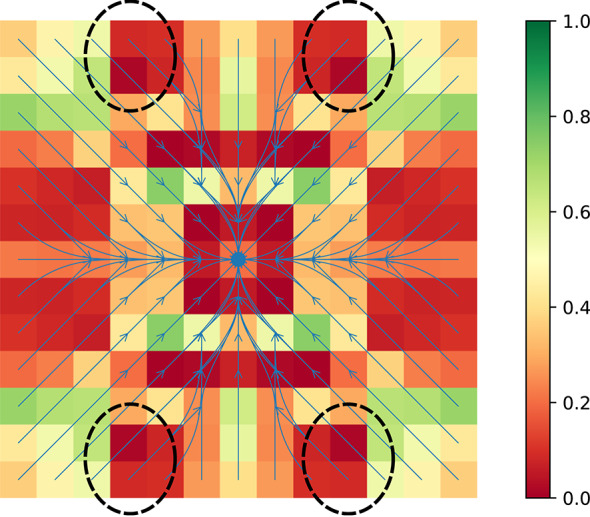
Close tie-breaking. To understand where the policy length choices are nearly arbitrary (and potentially numerically sensitive), we evaluate the value differences to runner-up actions of different lengths. These are shown in the plot for the $N=3$ policy of Fig. 10a, with notable artifacts circled, these being regions with close tie-breaking. The difference is expressed as a relative value, with green being a difference comparable to the values themselves and red being no difference, with a linear scale between

The plots showing policies (e.g., Figure 10a or 11) have a general pattern: sequences grow in length with increasing error, the error being visually apparent as a radial distance from the center of the plot. Evidently this pattern does not hold entirely consistently, however, and some examination of the cause of the non-radial nature and the unusual vertical gray states forming bars along the boundaries (see the dashed ellipses in Fig. 16 highlighting those states) is warranted.

Two aspects contribute in order to explain the artifacts. Firstly, from the transition dynamics: as Fig. 9 shows, the transition dynamics for the joint actions cover coarse shapes due to discretization, which shows up in the general ‘blocky’ nature of the policy. Secondly, specific artifacts like the vertical bars are the result of actions being ranked so closely together in value, that it is nearly arbitrary which one is picked. To see this, examine Fig. 16 showing the difference in value between the optimal policy action and the next-best action of a different length is shown. We see that when the difference is low (red), the policy is essentially indifferent to the length and selects it nearly arbitrarily, which leads to the jagged artifacts on the color map.

**Fig. 17:**
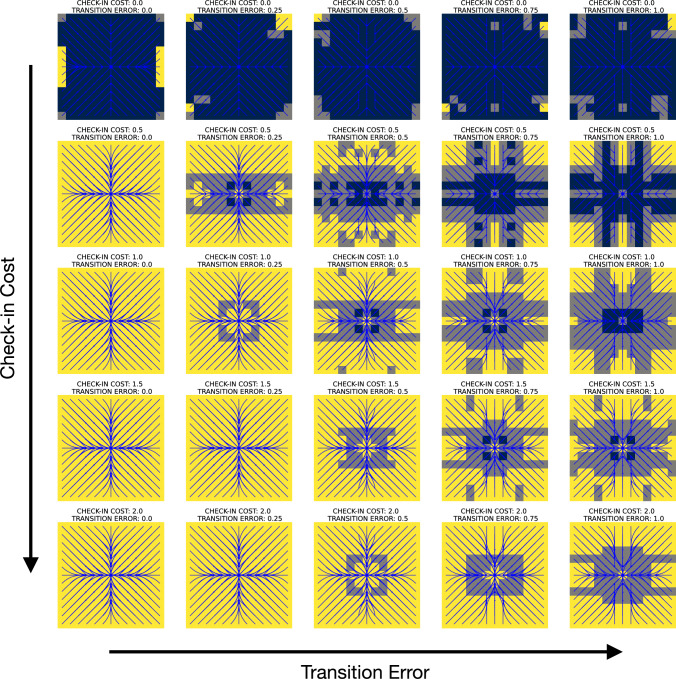
Balancing of check-in costs and uncertainty. Computed policies for various check-in costs and transition uncertainties. The element at position (4, 4) with check-in cost 1.5 and transition error 0.75 corresponds to Fig. 10a. The color scheme for action sequence lengths again maintains the conventions of earlier figures, i.e., navy=1/gray=2/yellow=3

#### Check-in cost and transition uncertainty

Further interesting relationships appear when one examines how differing parameters impact the policy, varying from low to high check-in costs and perfect to imperfect transitions, as visualized in Fig. 17.

With no check-in cost in the top row, the agents observe each other at every step (the artifacts here are also from numerically close values). With absolutely perfect transitions in the left column, the agents have no need for frequent check-ins and opt for as few as possible. As one proceeds from the left to the right with higher entropy transitions, the yellow of longer actions is replaced by the gray and blue of shorter actions as more frequent check-ins are needed to correct for those imperfect actions. Conversely, going downwards with costlier observations, actions lengthen as the agents take more risk to mitigate the cost of check-ins.

## Non-idealities

**Fig. 18:**
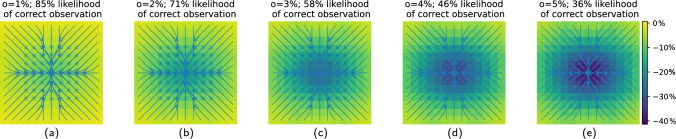
Impact of inconsistent observations. An evaluation of how increasing uncertainty in the observation model decreases state values. Here, in the formation-keeping problem, the pair of agents may obtain measurements along- and cross-track readings which disagree, as a function of parameter *o*

The models presented throughout this paper make three key assumptions about state observations, action selection, and action execution. Firstly, that, when an agent receives an observation of the state, the observation is perfect—indeed, the assumption of perfect state observability is central to ensuring coordination between the agents. Secondly, that agents receiving the same state observation select the same action, which requires a consistent tie-breaking mechanism. Thirdly, that all agents will take the same, deterministic amount of time to perform their portion of an action and observe the system state, resulting in coordinated observations at pre-scheduled times. While tie-breaking consistency is straightforward to implement, the assumptions about state observations and action execution can be violated in practice. When mutual state observations are performed by sensing and not via communication, as is the case in the hardware experiments in Sect. 5, different agents may reach different conclusions as to the state and, hence, execute mismatched actions. Different agents may also vary in the time to perform an action followed by a joint observation; the time may depend on the state and action themselves (e.g., farther agents may require extra time to observe each other or to communicate).

**Fig. 19:**
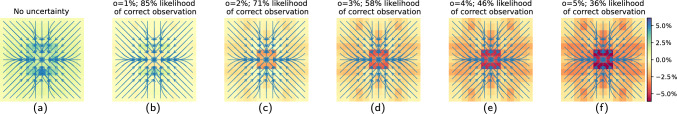
Policy quality under inconsistent observations. We visualize the difference between the state value of the optimal policy that ignores the possibility of mismatched observations and the state value achieved by a policy optimized for the $o=5\%$ case of Fig. 18e. (The former is just $\mathop {\mathrm {arg\,min}}\limits \mathrm {U_{{\!}_0}}$, the latter was obtained via brute-force enumeration of policies). The difference in values obtained by the policies is well under 10%

**Fig. 20:**
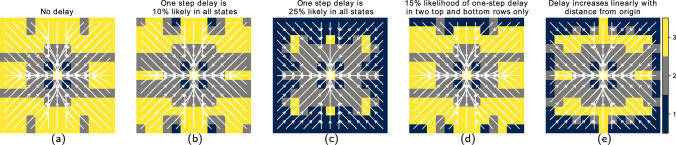
Policies when check-ins incur state-dependent delay. We compute optimal policies with different expected check-in delays, where **a** corresponds to Fig. 10a. The policy length is shown visually with colored cells employing the convention of earlier figures with navy=1/gray=2/yellow=3

In this section, we show how, under mild assumptions, the impact of the first effect can be quantified; and the second explicitly accounted for in policy design. We present numerical results based on the policy used in the hardware experiments in Sect. 5; but we stress that the proposed approaches to quantifying non-idealities are applicable to the general form of Problem 1.

### Inconsistent state observations

The assumption that, at check-in, all agents have access to a perfect observation of the joint system state can be violated in real systems with noisy sensors and unreliable communication. This is readily observed in our hardware experiments: in Fig. 13 the measurement error, which reports the mismatch between the agents’ observations, is consistently non-zero. Indeed, the measurement error is often of the same order of magnitude as the overall error experienced by the agents, suggesting that measurement inconsistencies may be a primary driver of error in the system. Agents then risk obtaining different macro–joint actions from the policy and executing mismatched individual actions, including ones with differing durations, which can misalign the agents’ future check-in times.

Two additional assumptions can address this. First, that each agent has a (potentially costly) ‘holding’ action which waits for others to conclude their own actions, so all agents can measure the system state at the same time. If the system dynamics present no drift, a no-op action (with no cost or effect on the system) is suitable. Second, we assume that agents have a synchronization mechanism that indicates when all agents have concluded their actions followed by an observation. In practice, this is not overly restrictive: if agents communicate to assess the system state, an acknowledgement mechanism suffices; and, if sensor observations are used to measure the shared state, agents can use in-band signaling (e.g., a colorful LED visible to other agent’s cameras). Importantly, the mechanism never requires an agent to communicate outside of its check-in times.

Under these assumptions, the effect of inconsistent observations on state value can be quantified, provided an observation model for the agents. Specifically, suppose a model $\mathrm{Pr}({\tilde{s}}_1 \!\ldots {\tilde{s}}_i \!\ldots {\tilde{s}}_n | s)$ characterizes the likelihood that agent *i* observes system state ${\tilde{s}}_i$ while the system is in state *s*. Note that the subscript *i* refers to agent *i*’s observation of the *overall* system state—the concatenation of agents’ individual states.

Upon observing state ${\tilde{s}}_i$, each agent *i* will select the macro–joint action given by $\mathop {\mathrm {arg\,min}}\limits \mathrm {U_{{\!}_0}}({\tilde{s}}_i)$ and execute its portion: $a^{{\!\!}^{{(\hspace{-0.4pt}i \hspace{-0.4pt})}\!}}_1({\tilde{s}}_i) \!\ldots a^{{\!\!}^{{(\hspace{-0.4pt}i \hspace{-0.4pt})}\!}}_m({\tilde{s}}_i)$, where we make explicit the dependency of actions on the measured state ${\tilde{s}}_i$. The resulting macro–joint action executed in state *s* is then

$$\begin{aligned} \tilde{\mathbf{a}}(s) = \begin{pmatrix} {\boldsymbol{\langle }}{a^{{\!\!}^{{(\hspace{-0.4pt}1 \hspace{-0.4pt})}\!}}_1({\tilde{s}}_1) {\boldsymbol{|}}{\cdots }{\boldsymbol{|}}a^{{\!\!}^{{(\hspace{-0.4pt}i \hspace{-0.4pt})}\!}}_1({\tilde{s}}_i) {\boldsymbol{|}}{\cdots }{\boldsymbol{|}}a^{{\!\!}^{{(\hspace{-0.4pt}n \hspace{-0.4pt})}\!}}_1({\tilde{s}}_n)}{\boldsymbol{\rangle }} \\ \vdots \\ {\boldsymbol{\langle }}{a^{{\!\!}^{{(\hspace{-0.4pt}1 \hspace{-0.4pt})}\!}}_m({\tilde{s}}_1) {\boldsymbol{|}}{\cdots }{\boldsymbol{|}}a^{{\!\!}^{{(\hspace{-0.4pt}i \hspace{-0.4pt})}\!}}_m({\tilde{s}}_i) {\boldsymbol{|}}{\cdots }{\boldsymbol{|}}a^{{\!\!}^{{(\hspace{-0.4pt}n \hspace{-0.4pt})}\!}}_m({\tilde{s}}_n)}{\boldsymbol{\rangle }} \end{pmatrix}^{\!\textsf {T}} \end{aligned}$$

with probability $\mathrm{Pr}({\tilde{s}}_1 \!\ldots {\tilde{s}}_i \!\ldots {\tilde{s}}_n | s)$. The individual agents’ actions may differ in length; in that case, agents which end early perform the holding action until all are ready to check in.

Figure 18 shows state values changing as uncertainty of the observation model increases for the problem in Sect. 5. Each robot has likelihood *o* of observing one of the eight neighbors of the system state *s*; the robots’ observations are independent, and the likelihood that both robots observe the correct state is thus $(1-8o)^2$.

As observation uncertainty increases, state values decrease significantly, especially in the central region of the state where the policy is less spatially uniform. To further investigate this phenomenon, we computed a policy that is locally optimal for the $o=5\%$ case (i.e., no single change to an action can improve the policy) through brute-force search, and compared the value of that policy with the value of the optimal policy (obtained via $\mathop {\mathrm {arg\,min}}\limits \mathrm {U_{{\!}_0}}$) across multiple levels of uncertainty. The result is shown in Fig. 19. The difference between the state value of the two policies is relatively small, well under 10%, even in presence of large observation noise; this suggests that the reduction in state value shown in Fig. 18 is due to the intrinsic complexity of the problem, and that the performance of the optimal policy is remarkably robust to observation noise.

#### Imperfect observation time

Equation (4) assumes that the next observation $s'$ is available to all agents simultaneously. In practice, measuring the joint state takes a finite amount of time that can depend on the system state and can differ for each agent. The state-dependent delay does not affect action costs; however, it does affect the cost-to-go through the discount factor $\mathbf {\gamma }(s,\mathbf{a})$ in Equations (1), (4), and (5).

This delay can be explicitly accounted for in planning with only a small adjustment. Assume that the time required for *all* agents to observe state *s* follows the distribution $\mathrm{Pr}(t_{\text {obs}}(s)=\tau )$. Then the discount in Equation (1) can be redefined as

$$\begin{aligned}\mathbf {\gamma }(s,\mathbf{a}) = \mathbb {E}_{\tau \sim \left( |\mathbf{a}|+ \mathrm{Pr}(t_\text {obs}(s))\right) } \left( \gamma ^\tau \right) ,\end{aligned}$$

and the resulting Bellman recursion solved for a policy that explicitly accounts for check-in delays. (The possibility of making this replacement is why $\mathbf {\gamma }(s,\mathbf{a})$ was defined as a separate function, being suggestive of a level of indirection).

Four cases of check-in delay are presented in Fig. 20 for visual comparison to the no-delay case (the latter appearing in Fig. 20a). Observe that action length generally decreases in regions with larger expected delays. In the example, state values are generally negative; accordingly, as the discount increases, the expected cost-to-go becomes less negative, making additional observations comparatively more attractive.

#### Imperfect action execution time

Finally, we discuss when agents may take different durations to execute their part of a macro–joint action. In the general case, Constructions 1 and 2 are no longer directly applicable, since one agent’s $i^{\text {th}}$ action may act on the system at the same time as another agent’s $j^{\text {th}}$ action. A continuous-time treatment becomes necessary to capture the resulting transition distribution and expected reward.

However, if the robots’ dynamics have no couplings (i.e., a robot’s actions only affect the robot’s own state, and the cost of a joint action is the sum of each agent’s individual action costs); and the system admits a no-op action where robots finishing early can wait for others before a check-in with no cost; then a simpler treatment is possible. One can show that Construction 1 holds, since each agent’s action only affect the agent’s portion of the system state; and Construction 2 can be updated by replacing $\gamma ^{k}$ with the expected sum of the discount factors for the individual agent’s actions:

$$\begin{aligned} \mathbf{C} _{{\mathrm{exc}}}^{\gamma } \left( s,\mathbf{a}\right)&=\! \sum _{j=1}^{n} \sum _{k=0}^{|\mathbf{a}|-1} \Gamma (a^{{\!\!}^{{(\hspace{-0.4pt}j \hspace{-0.4pt})}\!}}_1 a^{{\!\!}^{{(\hspace{-0.4pt}j \hspace{-0.4pt})}\!}}_2 \!\ldots a^{{\!\!}^{{(\hspace{-0.4pt}j \hspace{-0.4pt})}\!}}_k ) \times \\&\hspace{-1.3cm} \begin{bmatrix} \displaystyle \sum \limits _{\begin{array}{c} s^{{\!\!}^{{(\hspace{-0.4pt}j \hspace{-0.4pt})}\!}}_1 \!\ldots s^{{\!\!}^{{(\hspace{-0.4pt}j \hspace{-0.4pt})}\!}}_{k+1}\in {S^{{\!\!}^{{(\hspace{-0.4pt}j \hspace{-0.4pt})}\!}}}\!\!\!\times \cdots \times \! {S^{{\!\!}^{{(\hspace{-0.4pt}j \hspace{-0.4pt})}\!}}} \\ \text {where } s^{{\!\!}^{{(\hspace{-0.4pt}j \hspace{-0.4pt})}\!}}_1 = s^{{\!\!}^{{(\hspace{-0.4pt}j \hspace{-0.4pt})}\!}} \end{array}} \hspace{-3.5ex} C_{{\mathrm{exc}}} (s^{{\!\!}^{{(\hspace{-0.4pt}j \hspace{-0.4pt})}\!}}_{k+1},a^{{\!\!}^{{(\hspace{-0.4pt}j \hspace{-0.4pt})}\!}}_{k+1}) \prod _{i=1}^{k} T(s^{{\!\!}^{{(\hspace{-0.4pt}j \hspace{-0.4pt})}\!}}_{i+1},a^{{\!\!}^{{(\hspace{-0.4pt}j \hspace{-0.4pt})}\!}}_i,s^{{\!\!}^{{(\hspace{-0.4pt}j \hspace{-0.4pt})}\!}}_i) \end{bmatrix} ,\\&\quad \text {using }\Gamma (a^{{\!\!}^{{(\hspace{-0.4pt}j \hspace{-0.4pt})}\!}}_1 a^{{\!\!}^{{(\hspace{-0.4pt}j \hspace{-0.4pt})}\!}}_2 \!\ldots a^{{\!\!}^{{(\hspace{-0.4pt}j \hspace{-0.4pt})}\!}}_k ) = \prod _{i=1}^{k}\mathbb {E}_{\tau \sim \mathrm{Pr}(|a^{{\!\!}^{{(\hspace{-0.4pt}j \hspace{-0.4pt})}\!}}_i|)}[\gamma ^{\tau }], \end{aligned}$$

with $\mathrm{Pr}(|a^{{\!\!}^{{(\hspace{-0.4pt}j \hspace{-0.4pt})}\!}}_i|)$ is the distribution of the duration of action $a^{{\!\!}^{{(\hspace{-0.4pt}j \hspace{-0.4pt})}\!}}_i$, agent *j*’s $i^\text {th}$ action in its macro-action.

The updated action costs and updated discount factor can then be used to compute the optimal policy, as was done for the case of imperfect observation time. Thus, for non-drift systems with decoupled agent dynamics, the effect of heterogeneous action execution times can be accounted for directly in policy design. It it hardly necessary to remark that among the examples considered in this paper, both the gliders and the Turtlebots satisfy these assumptions: their dynamics are decoupled, and both systems can stop and wait for the other agent to become available.

## Conclusion

This paper proposes an mdp formulation for multi-agent sequential decision-making problems where agents must decide not only what actions to perform, but also when to jointly observe the system state; such decisions are made during execution, but committing to a joint observation schedule in advance can be advantageous from a cost perspective. This is, thus, a jointly endogenous observation process and, since observations are modeled as bearing costs, optimal solutions often end up employing observations rather sparingly. We examine multiple versions of the problem, with different observation and rescheduling costs; we then study in more detail the two cases where rescheduling observations has infinite cost (effectively requiring the agents to commit to an observation schedule in advance), and where the cost of rescheduling is zero (which allows agents to re-negotiate the time of the next joint observation at every check-in). We find that, at least for small-scale problems, the approach is computationally tractable, and demonstrate its practicality on hardware for a stylized formation-keeping scenario. Having conducted an appraisal of the properties of the policies that are produced, we see that they appear effective even in the presence of noisy and delayed state observations.

A number of directions for future research are of interest. First, the action set $\mathcal {A}$ is a subset of all possible macro–joint actions, so one might explore how to efficiently prune this set to reduce the computational requirements of the problem, extending prior work (Rossi and Shell, 2022) that addressed fixed check-in times. Pruning can both remove macro–joint actions whose temporal prefixes are dominated, exploiting the temporal structure of the problem; and macro–joint actions that result by different individual agents’ actions, but map to the same transitions, exploiting the multi-agent nature of the formulation. Second, we plan to propose techniques to make the policy more robust to imperfect state observations, by penalizing highly inconsistent actions in neighboring (and therefore easily-confused) states. Thirdly, we plan to extend the formulation to capture selected *partial* state observations that can arise in multi-agent systems (e.g., improved knowledge of the agent’s own state, or Boolean-valued observations reporting whether the system state lies inside a given set), while retaining computational tractability. Finally, it would be interesting to explore decentralized planning as well, paving the way for online adaptation to learned system dynamics.

## Data Availability

No datasets were generated or analysed during the current study.
